# Understanding the initial events of the oxidative damage and protection mechanisms of the AA9 lytic polysaccharide monooxygenase family†

**DOI:** 10.1039/d3sc05933b

**Published:** 2024-01-09

**Authors:** Marlisa M. Hagemann, Erna K. Wieduwilt, Erik D. Hedegård

**Affiliations:** a Department of Physics, Chemistry, and Pharmacy, University of Southern Denmark Campusvej 55 5230 Odense Denmark erdh@sdu.dk

## Abstract

Lytic polysaccharide monooxygenase (LPMO) is a new class of oxidoreductases that boosts polysaccharide degradation employing a copper active site. This boost may facilitate the cost-efficient production of biofuels and high-value chemicals from polysaccharides such as lignocellulose. Unfortunately, self-oxidation of the active site inactivates LPMOs. Other oxidoreductases employ hole-hopping mechanisms as protection against oxidative damage, but little is generally known about the details of these mechanisms. Herein, we employ highly accurate theoretical models based on density functional theory (DFT) molecular mechanics (MM) hybrids to understand the initial steps in LPMOs' protective measures against self-oxidation; we identify several intermediates recently proposed from experiment, and quantify which are important for protective hole-hopping pathways. Investigations on two different LPMOs show consistently that a tyrosine residue close to copper is crucial for protection: this explains recent experiments, showing that LPMOs without this tyrosine are more susceptible to self-oxidation.

## Abstract 2

We employed a QM/MM model for two different LPMOs and show that the direct oxidation of a tyrosine is crucial for protection against oxidative damage, while the formation of a tyrosyl *via* a histidyl is generally not feasible.

## Graphical abstract

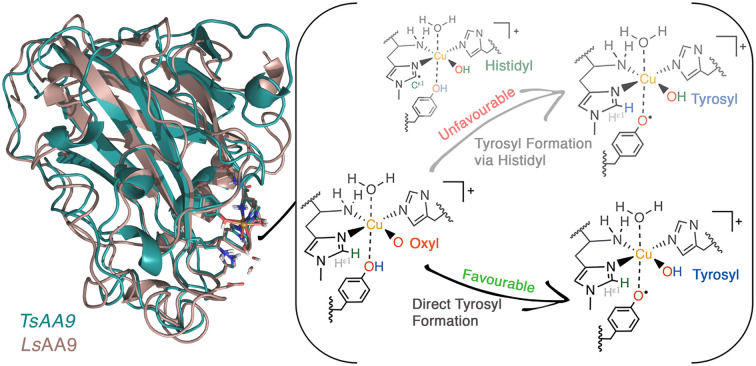

## Introduction

The heavy fossil fuel dependence of global energy production^1,2^ represents a major challenge in transitioning to a sustainable society. Further, essential products, like fertilizers, plastics, and chemicals, are also derived from fossil fuels,^3^ and transitioning will thus require alternative feedstocks.

**Fig. 1:**
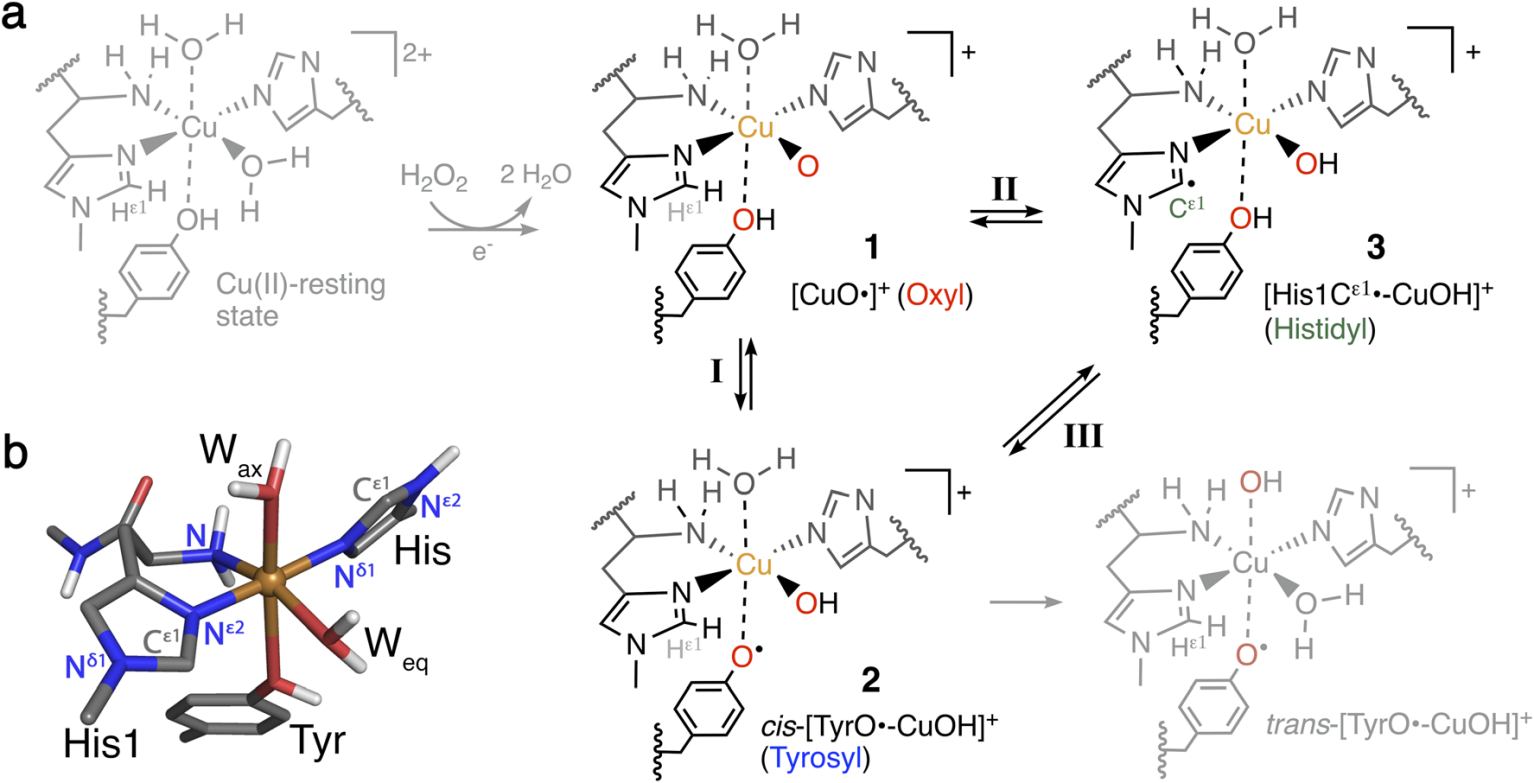
(a) Formation of Cu(ii)-oxyl 1 from the Cu(ii)-resting state (in gray) with a reductant and H_2_O_2_, and generation of intermediates 2 and 3 from 1. Intermediates 1–3 (in black) are investigated here for their role as part of oxidative damage or protective pathways (species in gray are not included in this study). (b) Ball-and-stick model of the first coordination sphere of the QM/MM optimized Cu(ii)-resting state of *Ls*AA9 employing TPSS/def2-SV(P).

Part of the solution may come from the production of fuel and chemicals from lignocellulose which comprise an abundant and cheap resource that does not compromise food security.^4–6^ Yet, the cost-efficient production of biofuels and materials or chemicals from lignocellulose is currently hampered by its natural recalcitrance.^7^ Consequently, the discovery of a new class of enzymes that boosts the degradation of polysaccharides has attracted considerable attention.^7–12^ This enzyme class is now termed lytic polysaccharide monooxygenase (LPMO) and is currently grouped into eight families: AA9 to AA11 and AA13 to AA17.^8,9,13–19^ The LPMOs differ in substrate-specificity^8,14–16,19–25^ but they generally catalyze the oxidation of the glycosidic C–H bonds in the polysaccharide, leading ultimately to a cleavage of the glycosidic bond.^10,11,26^ This cleavage causes the boosting effect and is administered by a copper-containing active site with Cu coordinated by two histidines^7,16–19,27^ that has been coined the histidine brace.^22^ The active site with the histidine brace in the Cu(ii)-resting state is shown in Fig. 1.

The oxidation of inactive C–H bonds requires a potent oxidant; several theoretical studies have shown that a Cu(ii)-oxyl^11,28,29^ is sufficiently potent. This Cu(ii)-oxyl can be formed with a suitable co-substrate after reduction of the Cu(ii)-resting state (see Fig. 1, structure 1). Whether this co-substrate is O_2_ or H_2_O_2_ is still an ongoing debate,^30,31^ although some LPMOs have been shown to use exclusively hydrogen peroxide.^32,33^

While a major focus in LPMO research has been the investigation of the substrate oxidation mechanism,^10,11,27,34^ the potent oxidative species can also cause oxidative damage to LPMOs in the absence of a substrate. This self-oxidation remains poorly understood but renders LPMOs inactive and thus hinders efficient exploitation of the enzymes. Proteomics techniques showed that oxidative damage is primarily confined to the two histidine residues coordinating the copper.^30^ We recently employed quantum-mechanics/molecular mechanics (QM/MM) to show that the oxidation of the histidine brace^35^ can be initiated by the hydrogen abstraction from either of the two histidines by a Cu(ii)-oxyl (1), forming a species (3) with a histidyl radical coupled to a [CuOH]^+^ moiety (see reaction II in Fig. 1). In a complementary series of experimental and theoretical investigations, we and others have proposed that a tyrosine close to the active site effectively protects LPMOs against the self-oxidation processes by forming a tyrosyl radical (2).^36–40^ Similar oxidative stress mechanisms have been previously studied employing DFT for Cu-amyloid beta peptide model complexes.^41^

The tyrosyl radical (2) can be formed by reaction I in Fig. 1 where the Cu(ii)-oxyl species (1) abstracts the hydrogen from the hydroxy group of the tyrosine,^40^ thus forming a [CuOH]^+^ moiety coordinated to a tyrosyl radical. Tyrosyl radicals have now been observed for a number of LPMOs and are characterized by having UV-vis spectra with strong, sharp absorption features around 400–420 nm.^36–38,42^ We note in passing that one investigation^39^ (for *Ls*AA9) showed rather different features, and based on QM/MM and time-dependent density functional theory (TD-DFT), we^40^ have argued that this spectrum originated in a different species (*trans*-[TyrO˙–CuOH]^+^ in Fig. 1).

The tyrosine residue has long mystified LPMO researchers: it is widely conserved in most LPMO families, except for the majority of AA10 LPMOs, where it is replaced by phenylalanine.^7,8,17,27,43^ It seems now that its role is to initialize a hole-hopping mechanism, known from other oxidoreductase enzymes: a radical species (“hole”) is generated close to the active site by a highly oxidizing species; this hole is then directed away (towards the surface) through electron-transfer chains comprised of aromatic/redox-active amino acids.^44^ Yet, detailed investigations of these reactions have been rare for oxidoreductases;^45,46^ and has only recently begun to appear for LPMOs.^36,37,39^ To confuse matters more, a hitherto undetected histidyl species was recently claimed to be observed through transient spectroscopy in *Ls*AA9.^42^ The histidyl radical was assigned based on UV-vis absorption at 360 nm and high-energy-resolution fluorescence-detected X-ray absorption spectroscopy (HERFD-XAS). It was argued that this radical was formed before the tyrosyl radical, but is otherwise part of the same protective hole-hopping mechanism. This investigation also detected a tyrosyl radical with its characteristic absorption features.

**Fig. 2:**
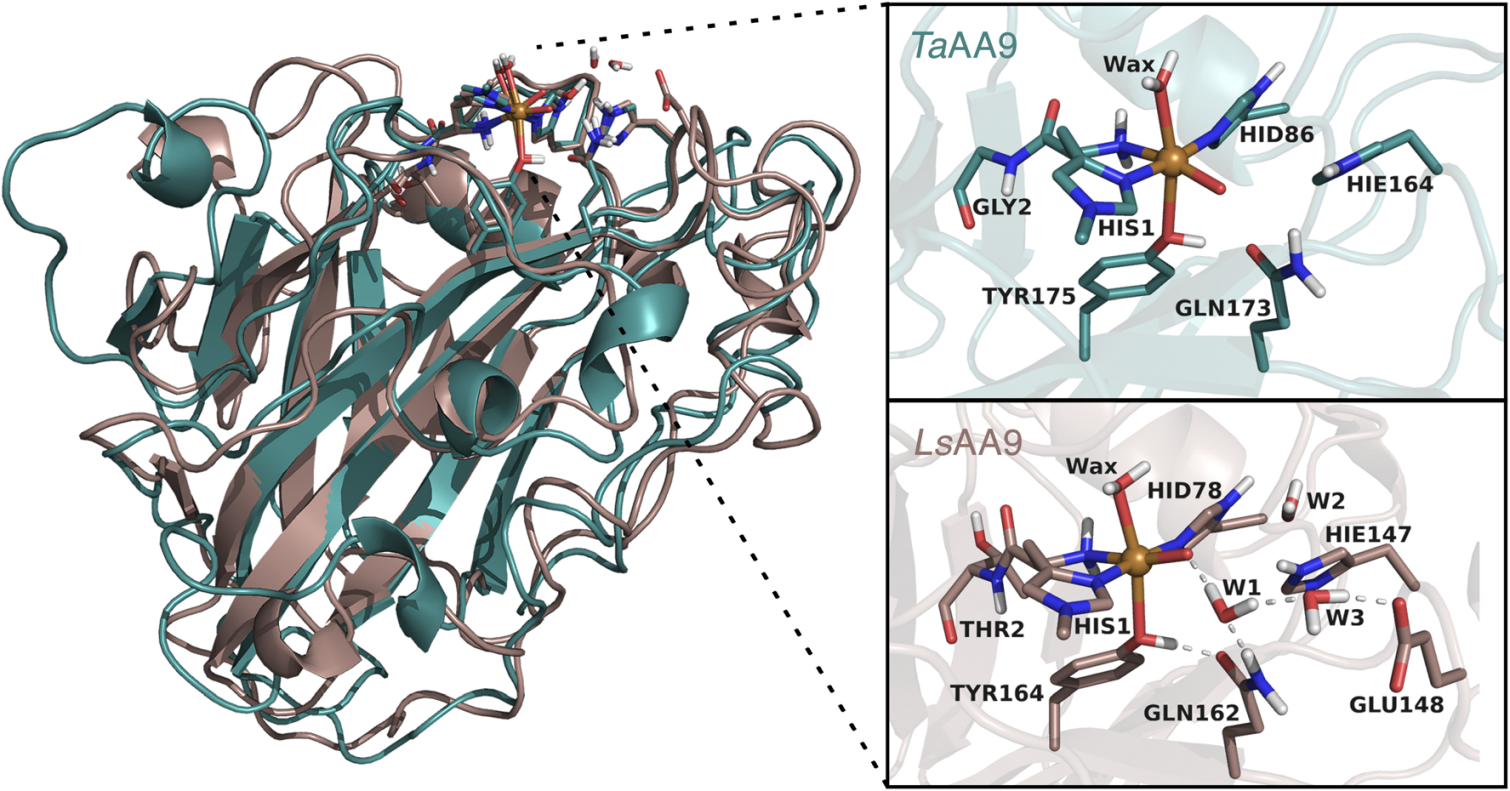
Comparison of the structures and active sites of *Ta*AA9 and *Ls*AA9. The structures are obtained from QM/MM optimizations for the oxyl [CuO]^+^. Labels refer to PDB 2YET^22^ and 5ACF^47^ for *Ta*AA9A and *Ls*AA9A, respectively. The QM region employed in this study are also shown (note that *Ta*AA9 has a similar hydrogen bonding network as *Ls*AA9 in the active site region, but this is kept in the MM region).

Several open questions now remain concerning the initial parts of the hole-hopping mechanism: (i) How does the tyrosyl radical formation compare energetically, relative to the histidyl radical formation? (ii) Are both tyrosyl and histidyl radicals part of the protective mechanism? (iii) Do different LPMOs display different mechanisms? The last open question arises since previous QM/MM calculations on the protective or oxidative damage mechanisms focused exclusively on a particular member of the AA9 family, namely *Ls*AA9,^35,40^ whereas experimental studies focused on different LPMOs.^36,39,42^ In this paper we will address (i) and (ii) by employing QM/MM calculations for reactions I–III in Fig. 1. We will also address (iii) by, for the first time, directly comparing oxidative damage and protective mechanisms of two enzymes, namely *Ls*AA9 and *Ta*AA9 (see Fig. 2). One of our findings is that *Ls*AA9 and *Ta*AA9 (that have quite similar second coordination spheres) occasionally display significant differences in their mechanisms. Given that little is known concerning the initial events of these protective mechanisms, our findings are likely to be highly relevant for other oxidoreductases as well.

## Computational details

### QM/MM calculations

All QM/MM calculations were based on equilibrated and QM/MM optimized structures of *Ls*AA9A from *Lentinus similis* (5ACF^47^) and *Ta*AA9A from *Thermoascus aurantiacus* (2YET^22^) from the studies ref. 35 and 48, respectively. We denote the two enzymes *Ls*AA9 and *Ta*AA9 in the following. A detailed description of the original protein setup, choice of protonation states and initial equilibration is given in ref. 28 and 29 for *Ls*AA9 and ref. 48 for *Ta*AA9.

The second-sphere tyrosine is present in both enzymes (Tyr175 and Tyr164 in *Ta*AA9 and *Ls*AA9, respectively, as shown in Fig. 2) and in both LPMOs a tyrosyl species was spectroscopically characterized.^36,42^ The protonation state of the second-coordination sphere histidine (His164) in *Ta*AA9 was changed from a doubly protonated form (Hip164) to a singly protonated one (Hie164), to match the protonation state present in the *Ls*AA9 structure.^40^

The employed QM regions are shown in Fig. 2. For *Ls*AA9 the QM region comprised the copper ion, the oxyl/hydroxyl ligand, the complete methylated His1 residue, and the side chains of His78, Tyr164, Thr2, Gln162, Glu148 and Hie147 as well as four water molecules. This is the same QM region also employed in ref. 35 and a slightly larger QM region (extended by Glu143 and three water molecules) as employed in ref. 40. For *Ta*AA9, the QM region consists of the copper ion and all the residues of the first coordination sphere, *i.e.*, the full His1 residue, the oxyl/hydroxyl ligand, the imidazole ring of His86, the phenol ring of Tyr175 and the axial water molecule. Additionally, the side chains of Gln173 and Hie164 were included, as well as parts of the backbone from the Gly2 residue. The latter was included up to the C^α^ atom and capped by replacing the carboxyl C with a hydrogen atom. The same applies to the corresponding residue Thr2 in *Ls*AA9.^49,50^ The side chains of all other residues were capped with a hydrogen by replacing C^α^. Note that *Ta*AA9 does not have a Glu residue corresponding to Glu148 in *Ls*AA9, and we therefore did not include the residue at this position in the QM region for *Ta*AA9. The QM region for *Ta*AA9 is larger than the one used in ref. 48 to allow better comparison to the one employed for *Ls*AA9. Notably, the active site water molecules were included in *Ls*AA9 as part of the QM region; there are also (non-coordinated) water molecules present in the second coordination sphere of *Ta*AA9, but we decided to keep them in the MM region as done in ref. 48. The non-coordinated water molecules are therefore accounted for in both *Ta*AA9 and *Ls*AA9, albeit at different levels of theory. Generally, the MM region only showed a small contribution to the QM/MM reaction energies (≤10 kJ mol^−1^) and thus we do not expect an explicit treatment of non-coordinated water molecules in the QM region of *Ta*AA9 to significantly change the energetics.

All structure optimizations and energy calculations employed a substractive QM/MM approach with electrostatic embedding as implemented in the modular program ComQum.^49,51^ ComQum interfaces the QM software Turbomole^52^ and the MM software AMBER.^53^ For all the structure optimizations the dispersion-corrected TPSS-D3 functional^54,55^ with Becke–Johnson damping^56^ and a def2-SV(P) basis set^57,58^ were employed. For all calculations with TPSS the resolution of identity (RI) approximation with standard auxiliary basis sets was applied. All atoms in the MM region were kept fixed during the geometry optimizations.

The reaction and activation energies were computed as linear transit calculations without thermochemical or zero-point vibrational energy corrections as these have shown to be small for hydrogen abstractions.^59–61^ The reported energies for the reactant, product and transition state structures were obtained from single-point calculations on the QM/MM optimized structures, employing the def2-TZVPP basis set^57^ (with an auxiliary basis set of the same size) and the functionals TPSS-D3 and B3LYP-D3.^55,62–64^ The single-point calculations include protein electrostatics, and an MM contribution calculated with TPSS/def2-SV(P). For brevity, we generally denote these functionals as TPSS and B3LYP throughout the paper although all calculations (for energies and geometries) always included dispersion corrections. On several occasions we compare to previous calculations,^35^ where generally TPSS/def2-SV(P) was used for geometry optimizations and B3LYP/def2-TZVPD for single point energies.

Our calculations took departure from the [CuO]^+^ species (1 in Fig. 1). This intermediate was selected as previous calculations have shown that this is the most likely species to abstract hydrogen from the substrate, and it is readily formed from the reaction between the reduced resting state and H_2_O_2_.^29,65–67^ From these previous calculations, we know that this [CuO]^+^ moiety is most accurately described as a Cu^2+^ and an O^−^˙ radical, spin-coupled to either a triplet or an open-shell singlet. Thus, the intermediates considered here are generally expected to attain either singlet and triplet spin states, and we have always attempted to obtain both of these spin states. As previous studies showed the closed-shell singlet states to be significantly higher in energy,^35,40^ the singlet states were only calculated as open-shell species in this study. The open-shell singlets were obtained in a spin-unrestricted (broken symmetry) formulation and the calculations were typically initiated from the triplet-state structures (using the triplet molecular orbital coefficients as initial guess). Similarly, for calculations with def2-TZVPP the open-shell singlet calculations were always started from the triplet molecular orbital coefficients. The convergence to an open-shell singlet was always confirmed by inspection of the Mulliken spin densities (which are reported in the ESI†). We compare the obtained electronic structure of 1 to previous calculations involving the Cu(ii)-oxyl (1) intermediate. However, since this has been discussed frequently in the literature, this discussion is moved to the ESI.†

For reaction II of *Ta*AA9 we were unable to locate an open-shell singlet for 3, although it could be obtained for a conformer of 3, which we here denote 3′. The calculations along reaction II collapse into a closed-shell singlet along the linear transit, the last open-shell singlet energy being 125 kJ mol^−1^ (distance between O_oxyl_ and H^OH^_Tyr_ restraint to 1.27 Å) for B3LYP and 84 kJ mol^−1^ (distance restraint to 1.30 Å) for TPSS, *cf.* Table S3.† This issue has also been observed previously for the TPSS functional with *Ls*AA9.^35^ In these cases, we base the discussion on the triplet state.

For the calculations of reaction III, we observed for both *Ls*AA9 and *Ta*AA9 that the QM/MM energies for the barrier showed high MM contributions (>33 kJ mol^−1^). We analyzed the energy contributions from individual residues and observed that this was caused by a residue close to the tyrosine (Phe43 and Pro30 in *Ls*AA9 and *Ta*AA9, respectively). Hence, we chose to include these residues in the QM region for this reaction, reducing the MM contributions to ∼13 kJ mol^−1^ for both enzymes. Since the reaction for *Ta*AA9 was more favorable for the triplet state and no open-shell singlet could be located for *Ls*AA9, we base the discussion on the triplet state.

### UV-vis spectra

UV-vis spectra were calculated for intermediates 2 and 3/3′ considering both open-shell singlet and triplet spin states, if open-shell singlet states were obtained. We performed TD-DFT calculations in Gaussian 16,^68^ employing the CAM-B3LYP functional^69^ and def2-TZVPP basis set (both as implemented in Gaussian) and including 45 states (roots). All UV-vis spectra were calculated from structures with the smaller QM regions (*i.e.*, calculations for 3′ and 3 do not include Phe43 and Pro30 for *Ls*AA9 and *Ta*AA9, respectively). For a better comparison to the spectra calculated for *Ls*AA9 in ref. 40, the input structures for *Ls*AA9 and *Ta*AA9 in the TD-DFT calculations were slightly truncated compared to the QM system that was employed for the structure optimizations and energy calculations. For *Ls*AA9, Thr2 was removed to the amide N, and for *Ta*AA9 only the amide-group of residue Gly2 was included. The cut bonds were saturated with a hydrogen atom (see Fig. S3 in the ESI†). Calculated oscillator strengths and energies were convoluted using a Gaussian function and a broadening factor of 0.3 eV. To prope the electrostatic effect of the environment, we in one case (for 2 of *Ta*AA9 in the triplet state) added point-charges from the QM/MM calculations.

## Results

We start by comparing *Ls*AA9 and *Ta*AA9 for the tyrosyl radical (2) formation directly from the Cu(ii)-oxyl (1) intermediate (*i.e.*, reaction I in Fig. 1). Next, we compare these two AA9 LPMOs for histidyl radical (3) formation (*i.e.* reaction II in Fig. 1). We finally discuss whether intermediates 2 and 3 can be inter-converted (reaction III in Fig. 1) as recently suggested.^42^

### Hydrogen abstraction from tyrosine

**Fig. 3:**
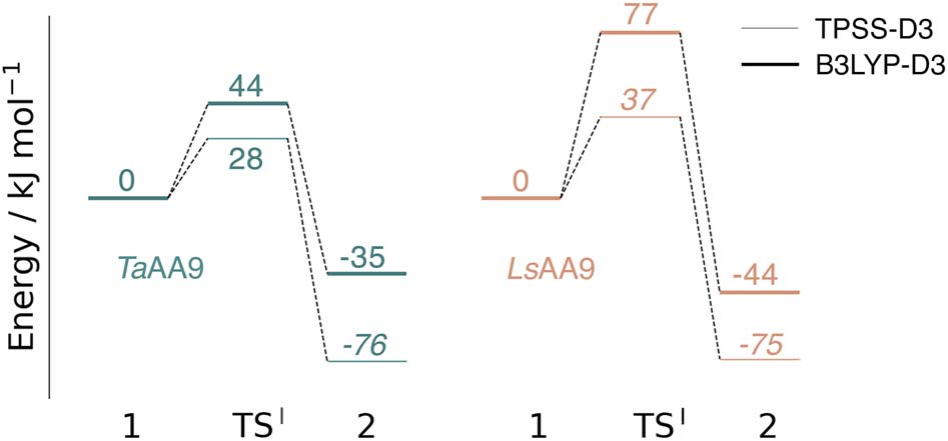
Energy diagrams (in kJ mol^−1^) of the reaction I for *Ta*AA9 (left) and *Ls*AA9 (right). Energies were obtained with def2-TZVPP based on structures optimized with TPSS/def2-SV(P). The reactant (1) in the triplet state was used as reference. Energies in bold refer to a triplet state and those in italic to open-shell singlet states.

**Fig. 4:**
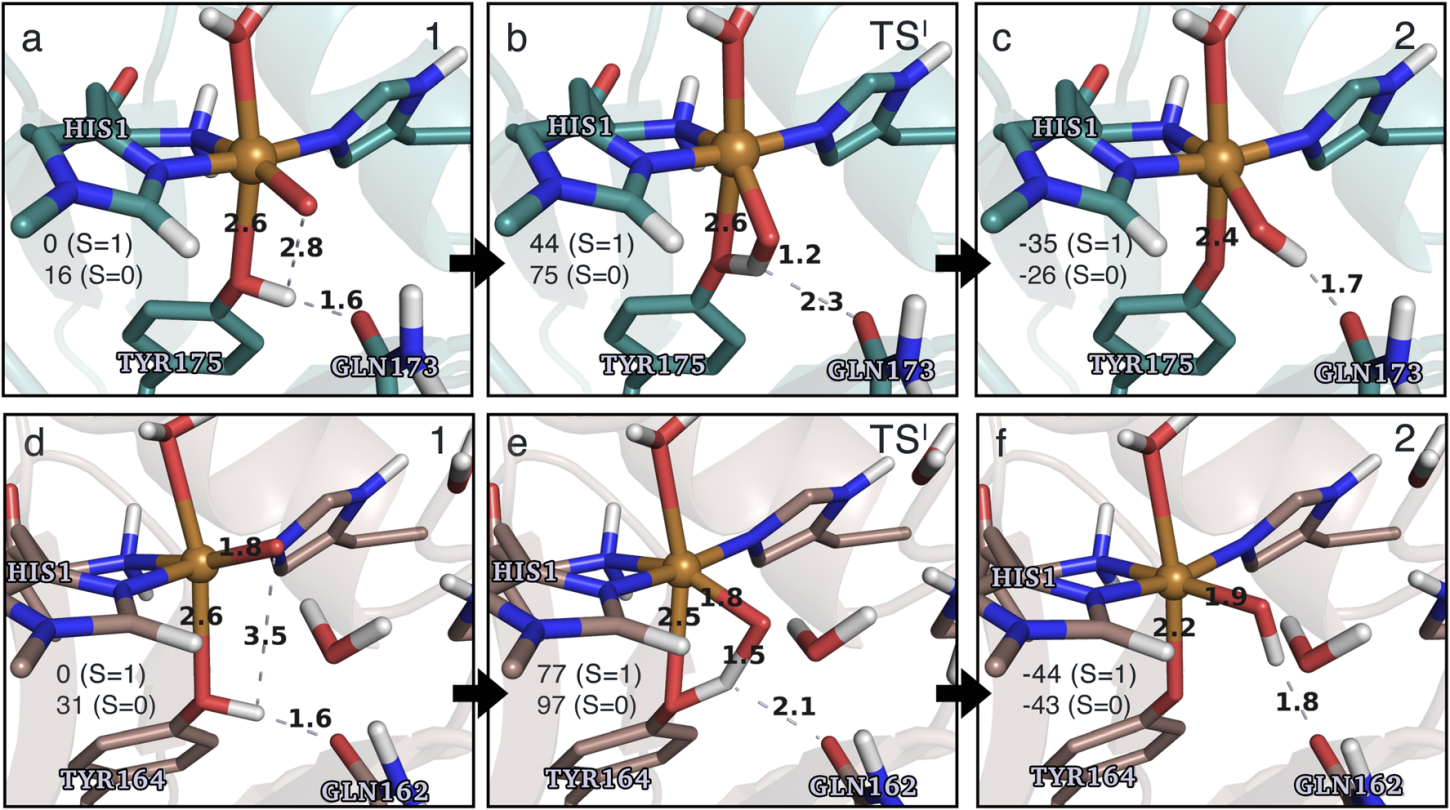
The hydrogen-abstraction reaction I illustrated for *Ta*AA9 (a–c) and *Ls*AA9 (d–f). The structures were optimized using TPSS/def2-SV(P) while the energies given were obtained employing B3LYP/def2-TZVPP. Only the structures for the most stable electron configuration (either open-shell singlet or triplet) are shown. Key distances are given in Å and energies in kJ mol^−1^ with reference to 1 in the triplet state. Further bond distances for the different intermediates can be found in Table S2.†

The calculated energy diagrams for reaction I of *Ta*AA9 and *Ls*AA9 are displayed in Fig. 3. We additionally show the structures and selected distances for the most stable electronic states in Fig. 4a–c (*Ta*AA9) and Fig. 4d–f (*Ls*AA9). A complete overview of reaction energies and barriers for both functionals is provided in Table S1.†

While there are some differences between the two employed functionals, both agree that the activation energies are consistently lower for *Ta*AA9: we obtain a barrier of 44 kJ mol^−1^ (28 kJ mol^−1^ with TPSS), compared to a reaction barrier of 77 kJ mol^−1^ for *Ls*AA9 (37 kJ mol^−1^ with TPSS). Both functionals also agree that the reaction is exothermic. In fact, the final reaction energies are quite similar between the two proteins, where we obtain an energy of −35 kJ mol^−1^ for *Ta*AA9 (−76 kJ mol^−1^ with TPSS) and −44 kJ mol^−1^ for *Ls*AA9 (−75 kJ mol^−1^ with TPSS). The result is qualitatively similar to previous calculations for *Ls*AA9 where a smaller QM region was employed;^40^ here the reaction barrier for reaction I was predicted to be 64 kJ mol^−1^ (53 kJ mol^−1^ with TPSS). We can thus conclude that *Ls*AA9 and *Ta*AA9 are likely to form the same tyrosyl intermediate, albeit with different kinetics. We can also compare structures of 1 and 2 as well as the transition state in the two LPMOs in Fig. 4. For both enzymes, the largest structural change between 1 and 2 is the change in the Cu–O_Tyr_ bond, which shortens from 2.6 Å (both enzymes) in 1 to 2.4 Å (*Ta*AA9) or 2.2 Å (*Ls*AA9) in 2. Thus, the de-protonation of tyrosine leads to coordination of the tyrosyl, consistent with a previous calculation on *Ls*AA9.^40^ In both enzymes, the tyrosine OH-group forms a hydrogen bond to a Gln residue in 1 (the bond distance is 1.6 Å in both *Ta*AA9 and *Ls*AA9). This hydrogen bond is partly broken in the transition state; here *Ta*AA9 and *Ls*AA9 are different as the bond distance is 2.3 Å in *Ta*AA9 and 2.1 Å in *Ls*AA9. Notably, the larger distance in *Ta*AA9 means that we can optimize a stable intermediate after breaking of the hydrogen bond to Gln, but this intermediate is close to degenerate with TS^I^, and we have therefore not included it in Fig. 4 (a full figure including this intermediate is given in the ESI, see Fig. S1 and S2†). Interestingly, the hydrogen bond to Gln is re-formed in 2 (with a distance of 1.7 Å in *Ta*AA9 and 1.8 Å in *Ls*AA9), where the OH group is now coordinated to Cu(ii). Other distances are roughly similar (*cf.* Table S2†), so the energetic differences between the reaction barrier of the two LPMOs in Fig. 3 may be traced back to the structural differences in the local hydrogen bonding of Gln to the transition state.

The spin states of 2 deserve a comment as several different results have been obtained in the literature: a somewhat noisy electron paramagnetic resonance (EPR) spectrum was recorded of 2 in *Ta*AA9,^36^ suggesting that a triplet is energetically within reach. Meanwhile Jones *et al.*^37^ obtained an EPR silent tyrosyl (2) for *Hj*AA9, while a very recent study proposes a triplet spin state for 2 in *Ls*AA9 (also based on EPR).^42^ We find that for *Ls*AA9, the singlet and triplet states are essentially degenerate with both functionals. Similarly, ref. 40 reported that the splittings for *Ls*AA9 are small (<5.5 kJ mol^−1^) for both functionals (the triplet state was found to be slightly more stable, independent of the functional used). Thus, for this LPMO our results are commensurate with the recent EPR results in ref. 42, *i.e.*, the triplet state is energetically within reach. The results for *Ta*AA9 are more ambiguous: the triplet state is most stable according to B3LYP (by 9 kJ mol^−1^), whereas TPSS predicts the open-shell singlet state to be more stable (by 20 kJ mol^−1^). The triplet is thus still within reach, but the magnitude of the splitting is more functional dependent. Obtaining the correct ordering of close-lying spin states is a known issue with DFT.^70^ However, with spin-state splittings of the size observed for 2, even highly correlated wave function methods may be challenged. For instance, Delcey *et al.*^71^ obtained spin-state splittings of 24 kJ mol^−1^ with restricted active space second-order perturbation (RASPT2) calculation for another metalloenzyme (a [NiFe]-hydrogenase), but were still unable to unequivocally assign the correct ground state. Meanwhile, our previous comparison to highly accurate multiconfigurational wavefunction (CASPT2) calculations on LPMO intermediates have also shown that discrepancies for spin-state splittings can occur with DFT.^72^ In conclusion, we can only expect qualitative results for reactions were 2 is involved (this is also the reason we mostly will base our conclusions on results obtained from two different DFT functionals).

In terms of spin densities (see Tables S7 and S8 in the ESI†), both *Ta*AA9 and *Ls*AA9 have significant spin density on tyrosine in 2. This was previously observed for *Ls*AA9 in ref. 40, and confirms that intermediate 2 observed in *Ta*AA9 and *Ls*AA9 have similar electronic structures. This electronic structure is a complicated coupling between the tyrosyl radical and a [CuOH]^+^ unit, where the latter itself is best described as Cu(ii) coupled to a OH radical. This complicated spin-coupling presumably also explains the observed differences between the two functionals. During reaction I, the spin density decreases on O_oxyl/hydroxyl_ and increases on tyrosine/tyrosyl. We also note that an in-depth analysis of the spin densities over the potential energy surface of reaction I for *Ls*AA9, reveals that for the TS^I^ state, TPSS obtains a broken-symmetry singlet with the expected spin distribution, but with the magnitude of the spin populations reduced (the issue is not seen for *Ta*AA9). This issue is similar to what was described in the Computational details and has also been observed previously,^35^ although the state obtained in Fig. 3 is not completely collapsed to a closed-shell singlet.

### Hydrogen abstraction from histidine

**Fig. 5:**
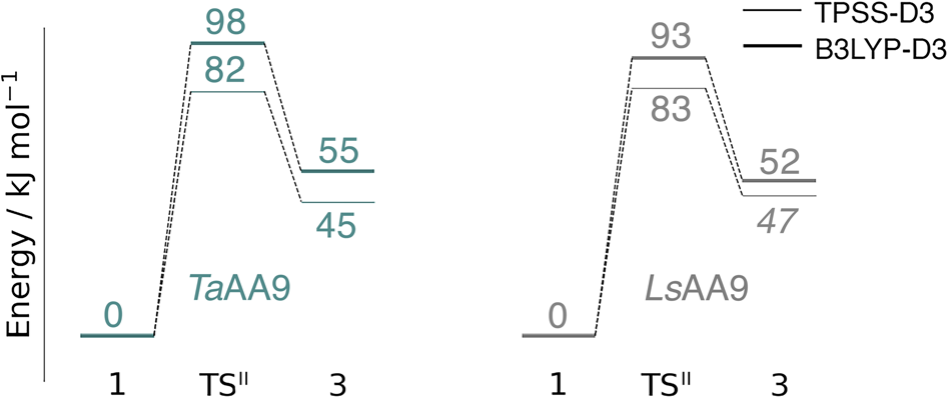
Energy diagrams (in kJ mol^−1^) of the reaction II for *Ta*AA9 (left) and *Ls*AA9 (right). Energies for *Ta*AA9 were obtained with def2-TZVPP based on structures optimized with TPSS/def2-SV(P). Energies for *Ls*AA9 are from ref. 35. The reactant (1) in the triplet state was used as reference. Energies in bold refer to a triplet state and those in italic to open-shell singlet states.

An alternative to the reaction investigated in the previous subsection is the abstraction of a hydrogen from the histidine brace by the [CuO]^+^ moiety in 1 (*i.e.*, reaction II in Fig. 1). Indeed, we recently investigated this for *Ls*AA9 as the first step of the oxidative self-damage reaction.^35^ We compare the reaction profiles (barrier and reaction energy) of reaction II for *Ta*AA9 and *Ls*AA9 in Fig. 5 (all calculated energies associated with reaction II are provided in Table S3†). As can be seen from Fig. 5, *Ls*AA9 and *Ta*AA9 overall have the same energy profile for the H-abstraction from the active site histidine (His1). The reaction barrier differs only by 5 kJ mol^−1^ (1 kJ mol^−1^ for TPSS) and the reaction energy differs by less than 3 kJ mol^−1^ for both functionals. Note that Fig. 5 does not include the highly exothermic OH recombination reaction investigated as part of the oxidative damage pathway in ref. 35 for *Ls*AA9. However, the similarity of the reaction energetics for the first part of the reaction for *Ls*AA9 and *Ta*AA9 suggest that the oxidative damage can proceed through this pathway, independent of the LPMO.

**Fig. 6:**
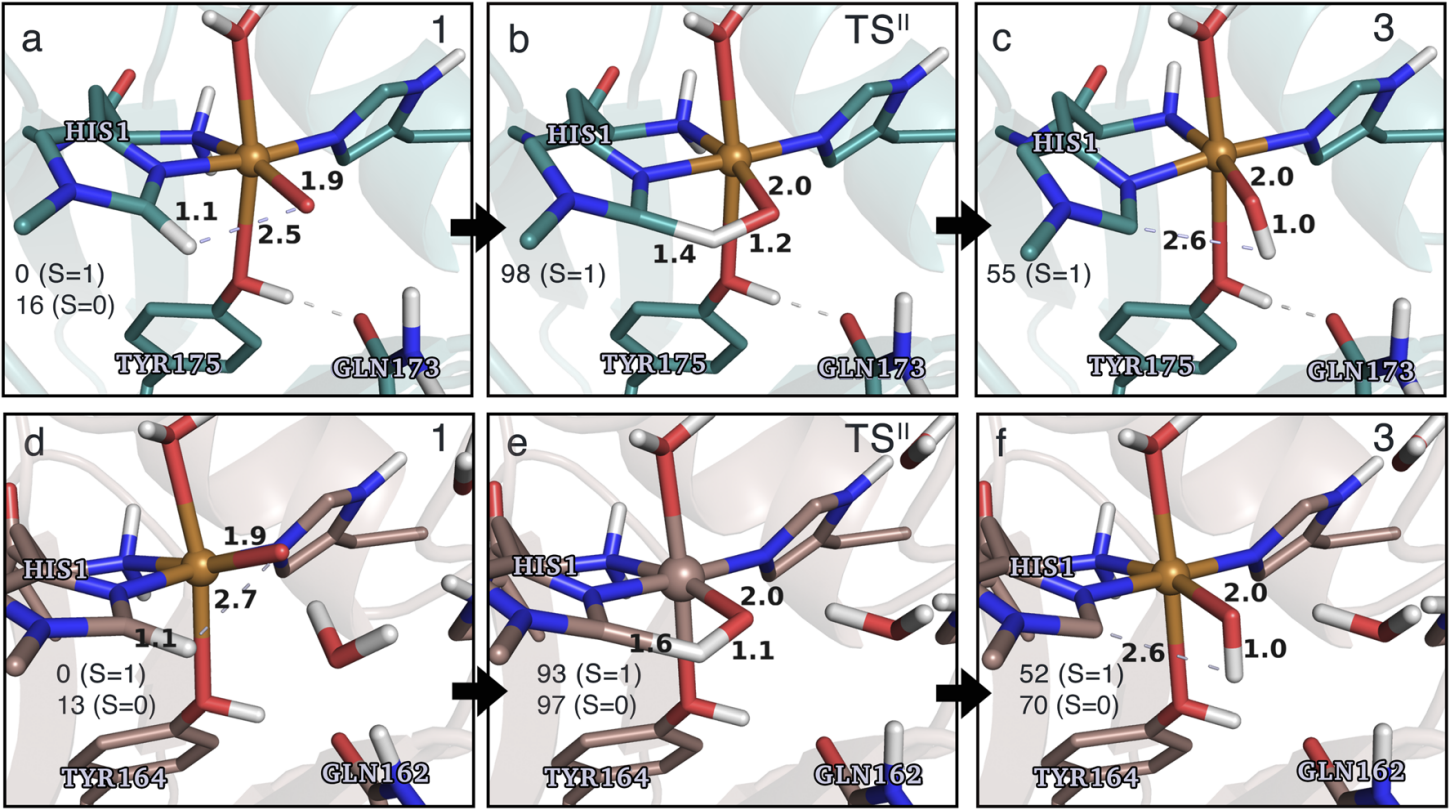
The hydrogen-abstraction reaction II illustrated for *Ta*AA9 (a–c) and *Ls*AA9 (d–f). The structures for *Ta*AA9 were optimized using TPSS/def2-SV(P) while the energies given were obtained employing B3LYP/def2-TZVPP. Structures and energies for *Ls*AA9 are from ref. 35. Only the structures for the most stable electron configuration (either open-shell singlet or triplet) are shown. Key distances are given in Å and energies in kJ mol^−1^ with reference to 1 in the triplet state. Further bond distances for the different intermediates of *Ta*AA9 can be found in Table S4.†

Structures for the reactants, transition states, and products, along with selected distances, are shown in Fig. 6 (additional distances are provided in Table S4 in the ESI†). As can be seen from this figure, structural changes during reaction II are similar for *Ta*AA9 and *Ls*AA9, as expected based on the similar reaction profiles: most copper–ligand bond distances undergo small changes with the sole exception being the Cu–O distance (see Table S4†), which increases by 0.09–0.10 Å, consistent with the protonation of the oxyl. We also note that for *Ta*AA9 we were able to obtain an isomer of 3, here denoted as 3′ (shown in Fig. 8a). This isomer differs from 3 in Fig. 6 in that the OH-group of the tyrosine points towards the C^ε1^ in the imidazole ring of His1. In 3, the OH group instead forms a hydrogen bond with the oxygen in Gln173. Since the energy difference between the isomers is rather small (Δ*E* = 12–19 kJ mol^−1^ in their triplet state depending on the functional), it is likely that they both exist in solution. The structure of the 3′ conformer seems optimal for the transfer of the H-atom of the tyrosine OH-group to the histidyl, according to reaction III in Fig. 1 as recently suggested.^42^ We investigate this possibility in the next subsection.

Based on the spin densities, differences between the electronic changes during reaction II are also minor between the two LPMOs: the spin densities (Table S7†) decrease significantly on oxygen in the OH group of the [CuOH]^+^ moiety in 3, compared to O_oxyl_ in [CuO]^+^ (1), while the spin density increases on His1. The increase mainly occurs on the de-protonated C^ε1^, suggesting that 3 is indeed a histidyl radical, coupled to a [CuOH]^+^ moiety. Similar to 2, this spin-coupling in 3 is also somewhat complicated. Yet, compared to the reaction I involving 2, the barrier and energetics of reaction II are less dependent on the employed functional, although the spin-state splitting are functional dependent, also for intermediate 3 (as will be detailed below). In the following subsection we will discuss another reaction (III) involving intermediate 2, where we also see a larger influence of the employed functional. Thus, reactions involving 2 seems to be particularly functional dependent, but more detailed investigations with multiconfigurational wave functions will be required to understand this difference.

In a recent experimental investigation with HERFD-XAS and UV-vis spectroscopy, a histidyl intermediate was claimed to be characterized as an open-shell spin singlet,^42^ although it is unclear how the spin state was determined. The histidyl intermediate was only characterized for *Ls*AA9 and we find the spin-state splitting for 3 in *Ls*AA9 to be small, but somewhat functional dependent: the splitting is only 5 kJ mol^−1^ with TPPS, the open-shell singlet being most stable. With the B3LYP functional we obtain a splitting of 18 kJ mol^−1^ with the triplet being most stable.^35^ For *Ta*AA9 the open-shell singlet calculations converged into a closed-shell singlet for 3, while we obtain both triplet and open-shell singlet states for the conformer 3′; in this case the open-shell singlet and triplet are essentially degenerate (the triplet is only 3 kJ mol^−1^ more stable with both TPSS and B3LYP). In light of the results for the tyrosyl radical (see previous subsection), it is likely that small differences in the active-site architectures between *Ta*AA9 and *Ls*AA9 lead to small differences in spin-state splittings. Since the splittings are small, this can also lead to differences in which spin states are most stable. However, with the present accuracy of the used functionals, the splittings are generally too small to clearly differentiate the spin states.

Before investigating reaction III, we note that we have here only investigated H-abstraction from His1. This was decided based on the structure in *Ta*AA9, where H-abstraction from His1 appeared more plausible compared to His86, given the notably shorter distance between H^ε1^ and O_oxyl_ (2.48 Å) in contrast to the H^ε1^ of His86 (3.39 Å). This was confirmed by a test calculation (Table S3†) for the triplet potential energy surface (PES), showing that the reaction barrier is indeed significantly higher compared to the abstraction from His1 (27–28 kJ mol^−1^ higher, depending on the functional). Moreover, the product is thermodynamically less stable by 19–21 kJ mol^−1^. It is interesting to note that in *Ls*AA9, the abstraction from His78 (equivalent to His86 in *Ta*AA9) occurs with only minor changes in energy (<8 kJ mol^−1^ for the reaction barrier and energy for both functionals), compared to the H-abstraction from His1.^35^ The difference between the two LPMOs occurs since the distances of H^ε1^ on His78 and His1 to O_oxyl_ in 1 is much closer (2.31 Å and 2.70 Å)^35^ in *Ls*AA9. This underlines that small differences in the active site architecture can lead to mechanistic differences, and this will become more apparent in the discussion regarding reaction III in the next subsection.

### Conversion of the histidyl radical to a tyrosyl radical

**Fig. 7:**
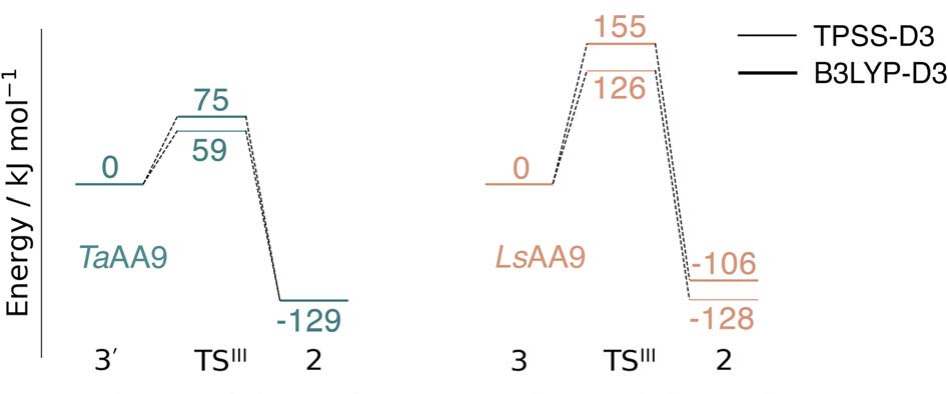
Energy diagrams (in kJ mol^−1^) for the reaction III for *Ta*AA9 (left) and *Ls*AA9 (right). The reactants 3′ and 3 for *Ta*AA9 and *Ls*AA9, respectively, were used as reference. Results were obtained for the triplet state with an extended QM region (see Computational details). Results for the smaller QM region are provided in Table S5 in the ESI.†

**Fig. 8:**
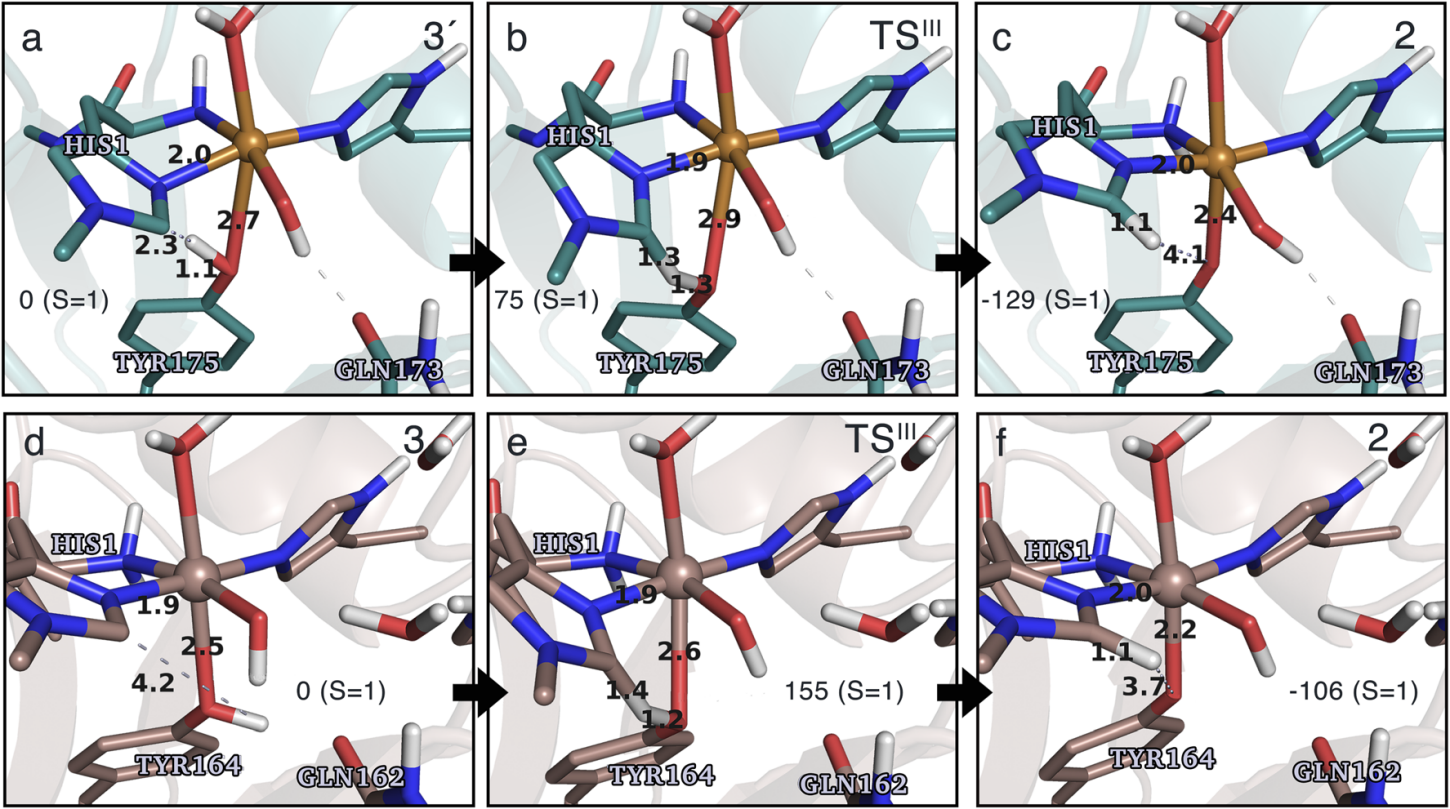
The hydrogen-abstraction reaction III illustrated for *Ta*AA9 (a–c) and *Ls*AA9 (d–f). The structures were optimized using TPSS/def2-SV(P) while the energies given were obtained employing B3LYP/def2-TZVPP. Key distances are given in Å and energies in kJ mol^−1^ with reference to the reactant. Further bond distances for the different intermediates can be found in Table S6.† Note that the structures were obtained with an extended QM region including Phe43 and Pro30 for *Ta*AA9 and *Ls*AA9, respectively.

Spurred by the recent proposal of a histidyl radical^42^ (3) as the first intermediate in a protective hole-hopping pathway, we investigated whether the histidyl radical (3) can abstract a hydrogen from the tyrosine, thereby restoring histidine while forming a tyrosyl radical (2). This reaction is labeled III in Fig. 1. The reaction barrier and energies are shown in Fig. 7 and 8. Interestingly, the two LPMOs are remarkably different: for *Ta*AA9 the reaction is kinetically feasible with a barrier of 75 kJ mol^−1^ (59 kJ mol^−1^ for TPSS), and is predicted to be thermodynamically favorable with a reaction energy of −129 kJ mol^−1^ for both functionals. In comparison, *Ls*AA9 shows a high reaction barrier of 155 kJ mol^−1^ (126 kJ mol^−1^ for TPSS) with a reaction energy of −129 kJ mol^−1^ (−128 kJ mol^−1^ for TPSS). The reactant, transition state, and product structures are shown in Fig. 8 (additional distances for chosen atoms are provided in Table S6 in the ESI†). The transition states also display somewhat different bond distances in the first coordination sphere to copper; the Cu–O_Tyr_ distance varies from 2.9 in *Ta*AA9 to 2.6 Å in *Ls*AA9, highlighting that the otherwise quite similar active-site architectures may lead to different reactivity. As for reaction I, the de-protonation of tyrosine's OH-group leads to a decrease in the Cu–O_Tyr175_ distance of 0.3 Å in the product (2). We finally note that we decided to use 3′ for *Ta*AA9 in Fig. 7 and 8. This choice was made since we then have consistently small MM energies for the barrier (the reaction energy is less affected) as the QM region was enlarged for 3′. We have not attempted to extend the QM region for 3, but based on the calculations with the smaller QM region, the energy difference between 3 and 3′ for *Ta*AA9 is sufficiently small so that none of the above conclusions change (see Table S5†).

## Discussion

**Fig. 9:**
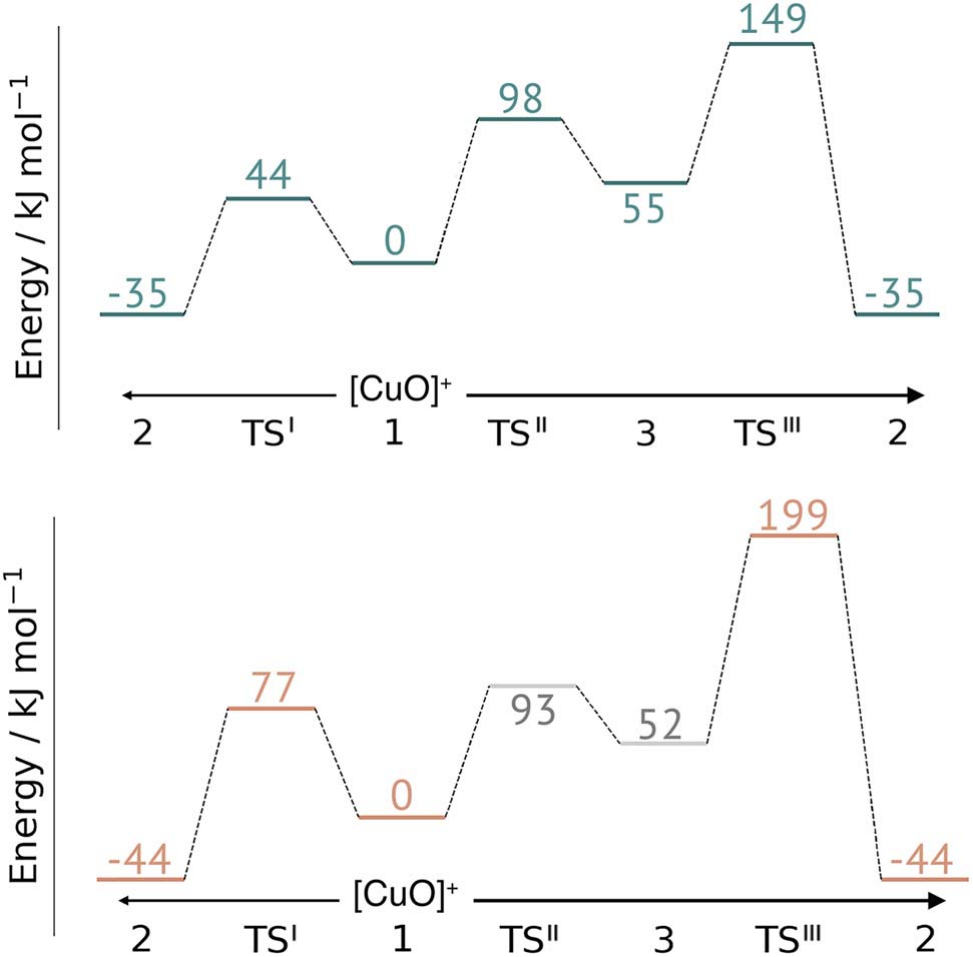
Comparison of the full reaction diagrams for different suggested protective mechanisms for *Ta*AA9 (top) and *Ls*AA9 (bottom) using the Cu(ii)-oxyl (1) as reference. Shown are only the most feasible energetics for B3LYP/def2-TZVPP (structures were optimized with TPSS/def2-SV(P)). Energies in gray are from ref. 35. Note that the reaction barrier TS^III^ was obtained with a bigger QM region.

This work represents the first comparison of oxidative damage and protective mechanisms in two different LPMOs: we have shown that it is energetically (and kinetically) feasible for *Ta*AA9 and *Ls*AA9 to form tyrosyl (2) radicals. This commensurates with experiments for both *Ta*AA9 and *Ls*AA9, were a number of studies detected 2 (ref. 36–38 and 42) for different LPMOs, including *Ta*AA9 (ref. 36) and *Ls*AA9.^42^ The detection of a histidyl radical (3) has only been proposed recently for *Ls*AA9.^42^ The histidyl radical was proposed to be part of a protective mechanism, where it was converted into the tyrosyl radical. Our results show that at least the formation of this radical is energetically similar for *Ta*AA9 and *Ls*AA9. However, if we consider the barrier for the conversion 3 → 2 alone (in Fig. 7), we find that for *Ls*AA9 the barrier is rather high (155 kJ mol^−1^ for B3LYP), but the overall reaction is feasible with a reaction energy of −106 kJ mol^−1^. It is interesting to compare this to the values obtained in ref. 35 where formation of 3 (from 1) is followed by recombination of the OH group from [ Cu–OH]^+^ in 2 to the histidyl radical, forming a 2-hydroxy-histidine (see Fig. 5 of ref. 35). This reaction was proposed to be part of the oxidative damage pathway and the barrier and reaction energy were calculated to be 49 kJ mol^−1^ and −287 kJ mol^−1^, respectively. The corresponding numbers for the overall reaction with the Cu(ii)-oxyl (1) as reference were calculated to be 101 kJ mol^−1^ for the barrier and −234 kJ mol^−1^ for the reaction energy.^35^ Clearly, this is still more favorable than the 199 kJ mol^−1^ and −44 kJ mol^−1^ in Fig. 9. Thus, the recombination reaction is more favorable than forming 3. We therefore speculate that if a histidyl radical is formed, it is more likely to lead to oxidative damage as suggested in ref. 35. We elaborate further on this in the following.

In Fig. 9 we compare different suggestions for protective mechanisms, illustrated by reactions I–III using the Cu(ii)-oxyl (1) as reference. Note that we have included results involving His78 (*Ls*AA9) and His86 (TaAA9) in the ESI (Tables S3 and S5†) but we will concentrate the discussion on His1 (this does not lead to any change of conclusions).

Reactions II and III were recently suggested as a protective hole-hopping mechanism.^42^ From Fig. 9, we can see that reaction I, *i.e.*, the direct formation of the tyrosyl radical (2) is generally preferable to reactions II and III, where the formation 2 goes through a histidyl radical (3): the overall barrier for forming 2*via*3 is 199 kJ mol^−1^ for *Ls*AA9, using [CuO]^+^ (1) as a reference (see Fig. 9). The barrier is lower for *Ta*AA9, but with 149 kJ mol^−1^, it must still be considered too high to be feasible. If a histidyl radical as 3 is formed, we therefore consider it more likely that it is formed in a competing reaction with formation of a tyrosyl radical (2). Notably, the high overall barrier may be a result of starting from the Cu(ii)-oxyl (1) species; we cannot exclude that another oxidative intermediate such as [Cu–OH]^2+^ or a free OH radical is responsible for initiating the oxidative damage, and we are currently investigating such alternatives.

In a broader perspective, our results provide a mechanistic explanation for that, similar to other oxidoreductases,^44^ the LPMO active-site tyrosine is critical for initializing the protective hole-hopping mechanism. This tyrosine is widely conserved in most LPMO families, except for the majority of AA10 LPMOs, where it is replaced by phenylalanine.^7,8,17,27,43^ Our results can thus explain that fungal AA9 LPMOs are less prone to oxidative damage than their bacterial (AA10) counterparts as experimental studies recently have discovered.^73^

In a previous paper^40^ we compared calculated UV-vis spectra with the characteristic observed bands around 400–420 nm for 2,^36–38,42^ but our calculations in ref. 40 were exclusively done on *Ls*AA9. With our QM/MM optimized structure of 2 in *Ta*AA9, we can now compare the corresponding UV-vis spectrum to the spectrum from *Ls*AA9 (see Fig. S4 in the ESI†). The calculated spectra are qualitatively similar displaying strong charge transfer transitions involving the tyrosyl radical close to 400 nm. The most intense transitions in *Ta*AA9 are shown at 357 nm (3.48 eV) for the triplet and 386 nm (3.22 eV) for the open-shell singlet. The latter transition shows excellent correspondence with the most intense peak of the calculated open-shell singlet of *Ls*AA9 at 388 nm (3.20 eV).^40^ Considering that we carried out the TD-DFT calculations as vacuum calculations, the intense peak from the open-shell singlet at 386 nm (3.22 eV) is in reasonable correspondence with the experimental room temperature absorption spectrum for *Ta*AA9 with the most intense peak at 420 nm (2.95 eV).^36,42^ The corresponding experimental value for *Ls*AA9 is 414 nm (2.99 eV)^42^ – other AA9 LPMOs show intense peaks in the same region (see ref. 37 and 38). For the triplet state of *Ta*AA9, we also tried to calculate the spectrum of 2 including the electrostatics the enzyme as point-charges, but the effect of the point-charges on the position of the intense charge-transfer transitions from tyrosyl is minimal (see Fig. S5 in the ESI†).

Since we could qualitatively reproduce the UV-vis spectra of 2, we additionally calculated the UV-vis spectrum of 3 for both *Ta*AA9 and *Ls*AA9 (Fig. S6 and S7 in the ESI†). Intermediate 3 has only been observed for *Ls*AA9 and the peak that experimentally is assigned the histidyl intermediate^42^ is obtained at 360 nm (3.44 eV). The TD-DFT calculations do predict intense transitions involving orbitals of histidyl character (further discussions are provided in the ESI†). However, these are at somewhat lower energies at 427 nm (2.91 eV) for the triplet and 406 nm (3.05 eV) for the open-shell singlet. The correspondence with the experimental values was clearly better for the tyrosyl radical (2). Intriguingly, the calculated spectrum for 3 in *Ta*AA9 is broader with two intense transitions at 342 nm (3.63 eV) and 395 nm (3.15 eV). Particularly, the former corresponds well to the observed band in *Ls*AA9, but we cannot presently explain why the calculated spectrum for *Ta*AA9 fits better with the experimental spectrum of *Ls*AA9. More benchmarks of the accuracy of TD-DFT – and preferably also investigations with multiconfigurational wave functions – will be required to confidently assign the spectrum of 3. Along these lines, Zhao *et al.*^42^ find that 17% exact exchange is optimal to reproduce relative intensities for HERFD-XAS spectra with TD-DFT, but we have refrained from attempting to re-parameterize the functional at this point.

## Conclusion

We have compared the initial steps of a protective hole-hopping mechanisms of two LPMOs, namely *Ta*AA9 and *Ls*AA9. The two investigated LPMOs have very similar active-site architectures, compared to other LPMOs (see, *e.g.*, ref. 7 and 34). We find similarities as well as remarkable differences in the protective mechanisms, highlighting that investigations on LPMOs as far as possible should consider several LPMOs. Regarding the similarities, our calculations show that the [CuO]^+^ moiety in the Cu(ii)-oxyl intermediate (1) for both *Ta*AA9 and *Ls*AA9 is capable of oxidizing tyrosine to a tyrosyl radical. The electronic structures of the formed tyrosyl radicals are overall similar in the two LPMOs, based on their spin densities and their calculated UV-vis spectra. The latter is commensurate with recent experimental investigations, with intense peaks around 400 nm.

We also find that the formation of a histidyl intermediate (3) has essentially the same reaction energy profile in the two LPMOs. However, it is more favorable energetically for both LPMOs to form the tyrosyl radical (2) than the histidyl radical (3). Overall, our results are commensurate with recent experiments showing that LPMOs without tyrosine are more susceptible to self-oxidation of the histidine brace.

The histidyl radical (3) has been proposed to be inter-converted to the tyrosyl radical (2), making it part of the protective hole-hopping mechanism. The above comparison between barriers and reaction energies for the formation of 3 and 2 cannot be reconciled with this mechanism. In fact, we find that the formation of a tyrosyl (2) *via* a histidyl (3) intermediate is generally not feasible with a reaction starting from a Cu(ii)-oxyl species (1). However, the LPMOs generally show quite different energetics regarding conversion between 3 and 2: for *Ta*AA9, the conversion (the barrier between 3 and 2) is feasible, and cannot be entirely ruled out, although it is unlikely with the presently employed oxidizing species (1). Meanwhile, this conversion for *Ls*AA9 seems not to be possible, regardless of the oxidizing species, since conversion between 3 and 2 has a very high barrier. Another remarkable difference between the LPMOs is that the barrier for formation of the tyrosyl radical (3) is generally larger for *Ls*AA9. A consequence of such differences may be that different LPMOs have different resistance toward oxidative damage. Investigation of other families (with and without a tyrosine residue close to copper) may reveal larger differences, and we are currently investigating this possibility.

## Data availability

Structures of all intermediates are deposited in a zenodo repository: https://doi.org/10.5281/zenodo.10495616.

## Author contributions

MMH: conceptualization, formal analysis, investigation (QM/MM), validation, visualization, writing – original draft, writing – review & editing; EKW: conceptualization, formal analysis, investigation (UV-vis), validation, participation in writing – original draft, writing – review & editing; EDH: conceptualization, formal analysis, funding acquisition, project administration, resources, supervision, validation, writing – review & editing.

## Conflicts of interest

There are no conflicts to declare.

## Supplementary Material

SC-015-D3SC05933B-s001

SC-015-D3SC05933B-s002

## References

[cit1] Ritchie, H.; Roser, M.; Rosado, P. Energy. Our World in Data 2022, https://ourworldindata.org/energy, accessed 2023-10-13

[cit2] bp Energy, Statistical Review of World Energy 2021, 2021, https://www.bp.com/content/dam/bp/business-sites/en/global/corporate/pdfs/energy-economics/statistical-review/bp-stats-review-2022-full-report.pdf, accessed 2023-10-13

[cit3] Levi P. G., Cullen J. M. (2018). Mapping global flows of chemicals: from fossil fuel feedstocks to chemical products. Environ. Sci. Technol..

[cit4] Chen H., Liu J., Chang X., Chen D., Xue Y., Liu P., Lin H., Han S. (2017). A review on the pretreatment of lignocellulose for high-value chemicals. Fuel Process. Technol..

[cit5] Chandel A. K., Garlapati V. K., Singh A. K., Antunes F. A. F., da Silva S. S. (2018). The path forward for lignocellulose biorefineries: bottlenecks, solutions, and perspective on commercialization. Bioresour. Technol..

[cit6] Binod P., Gnansounou E., Sindhu R., Pandey A. (2019). Enzymes for second generation biofuels: recent developments and future perspectives. Bioresour. Technol. Rep..

[cit7] Meier K. K., Jones S. M., Kaper T., Hansson H., Koetsier M. J., Karkehabadi S., Solomon E. I., Sandgren M., Kelemen B. (2017). Oxygen activation by Cu LPMOs in recalcitrant carbohydrate polysaccharide conversion to monomer sugars. Chem. Rev..

[cit8] Vaaje-Kolstad G., Westereng B., Horn S. J., Liu Z., Zhai H., Sørlie M., Eijsink V. G. (2010). An oxidative enzyme boosting the enzymatic conversion of recalcitrant polysaccharides. Science.

[cit9] Harris P. V., Welner D., McFarland K. C., Re E., Poulsen J.-C. N., Brown K., Salbo R., Ding E., Vlasenko H., Merino S., Xu F., Cherry J., Larsen S., Lo Leggio L. (2010). Stimulation of lignocellulosic biomass hydrolysis by proteins of glycoside hydrolase Family 61: Structure and Function of a Large, Enigmatic Family. Biochem..

[cit10] Beeson W. T., Vu V. V., Span E. A., Phillips C. M., Marletta M. A. (2015). Cellulose degradation by polysaccharide monooxygenases. Annu. Rev. Biochem..

[cit11] Walton P. H., Davies G. J. (2016). On the catalytic mechanisms of lytic polysaccharide monooxygenases. Curr. Opin. Struct. Biol..

[cit12] Johansen K. S. (2016). Discovery and industrial applications of lytic polysaccharide mono-oxygenases. Biochem. Soc. Trans..

[cit13] Hemsworth G. R., Henrissat B., Davies G. J., Walton P. H. (2014). Discovery and characterization of a new family of lytic polysaccharide monooxygenases. Nat. Chem. Biol..

[cit14] Vu V. V., Beeson W. T., Span E. A., Farquhar E. R., Marletta M. A. (2014). A family of starch-active polysaccharide monooxygenases. Proc. Natl. Acad. Sci. U. S. A..

[cit15] Lo Leggio L. (2015). *et al.*, Structure and boosting activity of a starch-degrading lytic polysaccharide monooxygenase. Nat. Commun..

[cit16] Couturier M. (2018). *et al.*, Lytic xylan oxidases from wood-decay fungi unlock biomass degradation. Nat. Chem. Biol..

[cit17] Sabbadin F. (2018). *et al.*, An ancient family of lytic polysaccharide monooxygenases with roles in arthropod development and biomass digestion. Nat. Commun..

[cit18] Filiatrault-Chastel C., Navarro D., Haon M., Grisel S., Herpoël-Gimbert I., Chevret D., Fanuel M., Henrissat B., Heiss-Blanquet S., Margeot A., Berrin J.-G. (2019). AA16, a new lytic polysaccharide monooxygenase family identified in fungal secretomes. Biotechnol. Biofuels.

[cit19] Sabbadin F., Urresti S., Henrissat B., Avrova A. O., Welsh L. R., Lindley P. J., Csukai M., Squires J. N., Walton P. H., Davies G. J., Bruce N. C., Whisson S. C., McQueen-Mason S. J. (2021). Secreted pectin monooxygenases drive plant infection by pathogenic oomycetes. Science.

[cit20] Langston J. A., Shaghasi T., Abbate E., Xu F., Vlasenko E., Sweeney M. D. (2011). Oxidoreductive cellulose depolymerization by the enzymes cellobiose dehydrogenase and glycoside hydrolase 61. Appl. Environ. Microbiol..

[cit21] Forsberg Z., Vaaje-Kolstad G., Westereng B., Bunæs A. C., Stenstrøm Y., MacKenzie A., Sørlie M., Horn S. J., Eijsink V. G. (2011). Cleavage of cellulose by a CBM33 protein. Protein Sci..

[cit22] Quinlan R. J. (2011). *et al.*, Insights into the oxidative degradation of cellulose by a copper metalloenzyme that exploits biomass components. Proc. Natl. Acad. Sci. U. S. A..

[cit23] Isaksen T., Westereng B., Aachmann F. L., Agger J. W., Kracher D., Kittl R., Ludwig R., Haltrich D., Eijsink V. G., Horn S. J. (2014). A C4-oxidizing lytic polysaccharide monooxygenase cleaving both cellulose and cello-oligosaccharides. J. Biol. Chem..

[cit24] Agger J. W., Isaksen T., Várnai A., Vidal-Melgosa S., Willats W. G., Ludwig R., Horn S. J., Eijsink V. G., Westereng B. (2014). Discovery of LPMO activity on hemicelluloses shows the importance of oxidative processes in plant cell wall degradation. Proc. Natl. Acad. Sci. U.S.A..

[cit25] Frommhagen M., Sforza S., Westphal A. H., Visser J., Hinz S. W., Koetsier M. J., van Berkel W. J., Gruppen H., Kabel M. A. (2015). Discovery of the combined oxidative cleavage of plant xylan and cellulose by a new fungal polysaccharide monooxygenase. Biotechnol. Biofuels.

[cit26] Forsberg Z., Sørlie M., Petrović D., Courtade G., Aachmann F. L., Vaaje-Kolstad G., Bissaro B., Røhr Å. K., Eijsink V. G. (2019). Polysaccharide degradation by lytic polysaccharide monooxygenases. Curr. Opin. Struct. Biol..

[cit27] Vaaje-Kolstad G., Forsberg Z., Loose J. S., Bissaro B., Eijsink V. G. (2017). Structural diversity of lytic polysaccharide monooxygenases. Curr. Opin. Struct. Biol..

[cit28] Hedegård E. D., Ryde U. (2017). Targeting the reactive intermediate in polysaccharide monooxygenases. J. Biol. Inorg. Chem..

[cit29] Hedegård E. D., Ryde U. (2018). Molecular mechanism of lytic polysaccharide monooxygenases. Chem. Sci..

[cit30] Bissaro B., Røhr Å. K., Müller G., Chylenski P., Skaugen M., Forsberg Z., Horn S. J., Vaaje-Kolstad G., Eijsink V. G. (2017). Oxidative cleavage of polysaccharides by monocopper enzymes depends on H_2_O_2_. Nat. Chem. Biol..

[cit31] Hangasky J. A., Iavarone A. T., Marletta M. A. (2018). Reactivity of O_2_ versus H_2_O_2_ with polysaccharide monooxygenases. Proc. Natl. Acad. Sci. U.S.A..

[cit32] Chang H., Gacias Amengual N., Botz A., Schwaiger L., Kracher D., Scheiblbrandner S., Csarman F., Ludwig R. (2022). Investigating lytic polysaccharide monooxygenase-assisted wood cell wall degradation with microsensors. Nat. Commun..

[cit33] Brander S., Tokin R., Ipsen J. Ø., Jensen P. E., Hernández-Rollán C., Nørholm M. H., Lo Leggio L., Dupree P., Johansen K. S. (2021). Scission of Glucosidic Bonds by a Lentinus similis Lytic Polysaccharide Monooxygenases Is Strictly Dependent on H_2_O_2_ while the Oxidation of Saccharide Products Depends on O_2_. ACS Catal..

[cit34] Hagemann M. M., Hedegård E. D. (2023). Molecular Mechanism of Substrate Oxidation in Lytic Polysaccharide Monooxygenases: Insight from Theoretical Investigations. Chem.–Eur. J..

[cit35] Torbjörnsson M., Hagemann M. M., Ryde U., Hedegård E. D. (2023). Histidine oxidation in lytic polysaccharide monooxygenase. J. Biol. Inorg. Chem..

[cit36] Singh R. K., Blossom B. M., Russo D. A., Singh R., Weihe H., Andersen N. H., Tiwari M. K., Jensen P. E., Felby C., Bjerrum M. J. (2020). Detection and characterization of a novel copper-dependent intermediate in a lytic polysaccharide monooxygenase. Chem.–Eur. J..

[cit37] Jones S. M., Transue W. J., Meier K. K., Kelemen B., Solomon E. I. (2020). Kinetic analysis of amino acid radicals formed in H_2_O_2_-driven CuI LPMO reoxidation implicates dominant homolytic reactivity. Proc. Natl. Acad. Sci. U. S. A..

[cit38] Hedison T. M., Breslmayr E., Shanmugam M., Karnpakdee K., Heyes D. J., Green A. P., Ludwig R., Scrutton N. S., Kracher D. (2021). Insights into the H_2_O_2_-driven catalytic mechanism of fungal lytic polysaccharide monooxygenases. FEBS J..

[cit39] Paradisi A., Johnston E. M., Tovborg M., Nicoll C. R., Ciano L., Dowle A., McMaster J., Hancock Y., Davies G. J., Walton P. H. (2019). Formation of a copper (II)–tyrosyl complex at the active site of lytic polysaccharide monooxygenases following oxidation by H_2_O_2_. J. Am. Chem. Soc..

[cit40] McEvoy A., Creutzberg J., Singh R. K., Bjerrum M. J., Hedegård E. D. (2021). The role of the active site tyrosine in the mechanism of lytic polysaccharide monooxygenase. Chem. Sci..

[cit41] Arrigoni F., Rizza F., Tisi R., De Gioia L., Zampella G., Bertini L. (2020). On the propagation of the OH radical produced by Cu-amyloid beta peptide model complexes. Insight from molecular modelling. Metallomics.

[cit42] Zhao J. (2023). *et al.*, Mapping the Initial Stages of a Protective Pathway that Enhances Catalytic Turnover by a Lytic Polysaccharide Monooxygenase. J. Am. Chem. Soc..

[cit43] Book A. J., Yennamalli R. M., Takasuka T. E., Currie C. R., Phillips G. N., Fox B. G. (2014). Evolution of substrate specificity in bacterial AA10 lytic polysaccharide monooxygenases. Biotechnol. Biofuels.

[cit44] Gray H. B., Winkler J. R. (2015). Hole hopping through tyrosine/tryptophan chains protects proteins from oxidative damage. Proc. Natl. Acad. Sci. U.S.A..

[cit45] Tommos C. (2022). Insights into the Thermodynamics and Kinetics of Amino-Acid Radicals in Proteins. Annu. Rev. Biophys..

[cit46] Gray H. B., Winkler J. R. (2021). Functional and protective hole hopping in metalloenzymes. Chem. Sci..

[cit47] Frandsen K. E. H. (2016). *et al.*, The molecular basis of polysaccharide cleavage by lytic polysaccharide monooxygenases. Nat. Chem. Biol..

[cit48] Hedegård E. D., Ryde U. (2017). Multiscale modelling of lytic polysaccharide monooxygenases. ACS Omega.

[cit49] Ryde U. (1996). The coordination of the catalytic zinc ion in alcohol dehydrogenase studied by combined quantum-chemical and molecular mechanics calculations. J. Comput.-Aided Mol. Des..

[cit50] Reuter N., Dejaegere A., Maigret B., Karplus M. (2000). Frontier bonds in QM/MM methods: A comparison of different approaches. J. Phys. Chem. A.

[cit51] Ryde U., Olsson M. H. (2001). Structure, strain, and reorganization energy of blue copper models in the protein. Int. J. Quantum Chem..

[cit52] Balasubramani S. G. (2020). *et al.*, TURBOMOLE: Modular program suite for ab initio quantum-chemical and condensed-matter simulations. J. Chem. Phys..

[cit53] Case D. A. , et al., Amber 2022, University of California, San Francisco. 2022

[cit54] Tao J., Perdew J. P., Staroverov V. N., Scuseria G. E. (2003). Climbing the density functional ladder: Nonempirical meta–generalized gradient approximation designed for molecules and solids. Phys. Rev. Lett..

[cit55] Grimme S., Antony J., Ehrlich S., Krieg H. (2010). A consistent and accurate ab initio parametrization of density functional dispersion correction (DFT-D) for the 94 elements H-Pu. J. Chem. Phys..

[cit56] Grimme S., Ehrlich S., Goerigk L. (2011). Effect of the damping function in dispersion corrected density functional theory. J. Comput. Chem..

[cit57] Schäfer A., Horn H., Ahlrichs R. (1992). Fully optimized contracted Gaussian basis sets for atoms Li to Kr. J. Chem. Phys..

[cit58] Eichkorn K., Weigend F., Treutler O., Ahlrichs R. (1997). Auxiliary basis sets for main row atoms and transition metals and their use to approximate Coulomb potentials. Theor. Chem. Acc..

[cit59] Wang B., Cao Z., Sharon D. A., Shaik S. (2015). Computations reveal a rich mechanistic variation of demethylation of N-methylated DNA/RNA nucleotides by FTO. ACS Catal..

[cit60] Senn H. M., Thiel S., Thiel W. (2005). Enzymatic hydroxylation in p-hydroxybenzoate hydroxylase: a case study for QM/MM molecular dynamics. J. Chem. Theory Comput..

[cit61] Senn H. M., Kaestner J., Breidung J., Thiel W. (2009). Finite-temperature effects in enzymatic reactions—Insights from QM/MM free-energy simulations. Can. J. Chem..

[cit62] Becke A. D. (1988). Density-functional exchange-energy approximation with correct asymptotic behavior. Phys. Rev. A.

[cit63] Becke A. D. (1993). Density-functional thermochemistry. III. The role of exact exchange. J. Chem. Phys..

[cit64] Lee C., Yang W., Parr R. G. (1988). Development of the Colle-Salvetti correlation-energy formula into a functional of the electron density. Phys. Rev. B: Condens. Matter Mater. Phys..

[cit65] Kim S., Ståhlberg J., Sandgren M., Paton R. S., Beckham G. T. (2014). Quantum mechanical calculations suggest that lytic polysaccharide monooxygenases use a copper-oxyl, oxygen-rebound mechanism. Proc. Natl. Acad. Sci. U.S.A..

[cit66] Wang B., Johnston E. M., Li P., Shaik S., Davies G. J., Walton P. H., Rovira C. (2018). QM/MM studies into the H_2_O_2_-dependent activity of lytic polysaccharide monooxygenases: evidence for the formation of a caged hydroxyl radical intermediate. ACS Catal..

[cit67] Bissaro B., Streit B., Isaksen I., Eijsink V. G., Beckham G. T., DuBois J. L., Røhr Å. K. (2020). Molecular mechanism of the chitinolytic peroxygenase reaction. Proc. Natl. Acad. Sci. U.S.A..

[cit68] Frisch M. J. , et al., Gaussian 16, Revision C.01, Gaussian Inc., Wallingford CT, 2016

[cit69] Yanai T., Tew D. P., Handy N. C. (2004). A new hybrid exchange–correlation functional using the Coulomb-attenuating method (CAM-B3LYP). Chem. Phys. Lett..

[cit70] Ghosh A. (2006). Transition metal spin state energetics and noninnocent systems: challenges for DFT in the bioinorganic arena. J. Biol. Inorg. Chem..

[cit71] Delcey M. G., Pierloot K., Phung Q. M., Vancoillie S., Lindh R., Ryde U. (2014). Accurate calculations of geometries and singlet-triplet energy differences for active-site models of [NiFe] hydrogenase. Phys. Chem. Chem. Phys..

[cit72] Larsson E. D., Dong G., Veryazov V., Ryde U., Hedegård E. D. (2020). Is density functional theory accurate for lytic polysaccharide monooxygenase enzymes?. Dalton Trans..

[cit73] Kuusk S., Eijsink V. G., Väljamäe P. (2023). The “life-span” of lytic polysaccharide monooxygenases (LPMOs) correlates to the number of turnovers in the reductant peroxidase reaction. J. Biol. Chem..

