# Supplementary material for: Understanding the initial events of the oxidative damage and protection mechanisms of the AA9 lytic polysaccharide monooxygenase family
Source: Chem Sci. 2024 Jan 9;15(7):2558–70. doi: 10.1039/d3sc05933b (PMC10866358; doi:10.1039/d3sc05933b)
Supplement: SC-015-D3SC05933B-s002 [file SC-015-D3SC05933B-s002.pdf]

Electronic Supplementary Material (ESI) for Chemical Science.
This journal is © The Royal Society of Chemistry 2024

# Supporting Information

**Spin-state splittings of intermediate 1:** The energies of the triplet (S = 1) and open-shell singlet (S = 0) for the Cu(II)-oxyl (**1**) intermediate are given in Table S1. For **1** in *Ta*AA9, the triplet is most stable, but the states are (as expected) close in energy with splittings of either 17 and 16 kJ/mol, depending on the functional. This is similar to what most previous studies have found.[34] For *Ls*AA9, the triplet and singlet are essentially degenerate with TPSS (the triplet is less stable, but only by 2 kJ/mol). With B3LYP we obtain a larger splitting of 31 kJ/mol, where the triplet is most stable. Small splittings were also obtained in ref. 35 with a splitting of 12-13 kJ/mol (the triplet being more stable), while ref. 40 obtained the triplet as the ground state but again with small splittings of 5 kJ/mol (TPSS) or 16 kJ/mol (B3LYP). The difference to these previous studies is either that we here use a slightly larger QM region[40] or a different functional in the structure optimization.[35] Notably, we also observe for *Ls*AA9 that TPSS gives an intermediate between the closed-shell and open-shell singlet, with a spin distribution similar to that of the B3LYP open-shell singlet but with 2-9 times lower magnitude and high spin contamination. Similar issues with spuriously low spin populations with TPSS were seen in some of our previous calculations, although it was less pronounced in the present calculations for **1** in *Ls*AA9.

For **1**, the spin densities (Tab. S7 and S8) overall fits with the expected triplet or singlet coupling between the unpaired electron of $d^9$ Cu(II) and the unpaired electron of the oxyl ($O^{\cdot -}$), although we occasionally observe small differences in spin-state splittings to previous calculations.[40 35]

**Energies and distances of all states associated with reaction I:**

Table S1: QM/MM energies (in kJ/mol) for *Ta*AA9 and *Ls*AA9 for reaction **I** in reference to the respective reactant $[CuO]^+$ (**1**) in the triplet state. Energies were obtained with def2-TZVPP based on structures optimized with TPSS/def2-SV(P).

| **Intermediate** | | **Energy difference** TPSS | B3LYP |
|---|---|---|---|
| ***Ta*AA9** | | | |
| **1** | (S=1) | 0.0 | 0.0 |
| **1** | (S=0) | 17.0 | 16.0 |
| $\mathbf{TS^{I*}}$[a] | (S=1) | 23.1 | 28.5 |
| $\mathbf{Int^{I}}$[a] | (S=1) | 21.2 | 28.9 |
| $\mathbf{TS^{I}}$ | (S=1) | 27.5 | 44.0 |
| $\mathbf{TS^{I}}$ | (S=0) | 37.4 | 74.5 |
| **2** | (S=1) | -56.0 | -35.1 |
| **2** | (S=0) | -75.9 | -26.2 |
| **2′** | (S=1) | -37.3 | -14.8 |
| **2′** | (S=0) | -57.9 | -14.9 |
| ***Ls*AA9** | | | |
| **1** | (S=1) | 0 | 0 |
| **1** | (S=0) | -1.6 | 31.3 |
| $\mathbf{TS^{I}}$ | (S=1) | 69.5 | 77.3 |
| $\mathbf{TS^{I}}$ | (S=0) | 36.9 | 96.8 |
| **2** | (S=1) | -73.1 | -44.3 |
| **2** | (S=0) | -74.7 | -42.9 |

[a] $\mathbf{TS^{I*}}$ and $\mathbf{Int^{I}}$ were only observed for the triplet state.

Table S2: Atom distances for chosen atoms in Å for the most stable electron configurations for reaction **I**. Structures were optimized using TPSS/def2-SV(P).

| **Intermediate** | | Distance $\text{Cu-O}_{\text{ox}}$ | $\text{Cu-N}^{\delta}_{\text{His1}}$ | $\text{Cu-N}_{\text{His1}}$ | $\text{Cu-N}^{\epsilon}_{\text{His86/78}}$ | $\text{Cu-O}_{\text{Tyr}}$ | $\text{H}^{OH}_{\text{Tyr}}\text{-O}_{\text{Gln}}$ | $\text{H}^{OH}_{\text{Tyr}}\text{-O}_{\text{ox}}$ |
|---|---|---|---|---|---|---|---|---|
| ***Ta*AA9** | | | | | | | | |
| **1** | (S=1) | 1.89 | 1.99 | 2.18 | 2.02 | 2.63 | 1.63 | 2.76 |
| $\mathbf{TS^{I*}}$ | (S=1) | 1.89 | 2.00 | 2.16 | 2.01 | 2.55 | 1.91 | 1.96 |
| $\mathbf{Int^{I}}$ | (S=1) | 1.91 | 1.99 | 2.18 | 2.00 | 2.68 | 2.62 | 1.60 |
| $\mathbf{TS^{I}}$ | (S=1) | 1.94 | 1.99 | 2.15 | 2.02 | 2.57 | 2.31 | 1.24 |
| **2** | (S=0) | 1.91 | 2.01 | 2.13 | 2.05 | 2.40 | 1.67 | 1.03 |
| **2′** | (S=0) | 1.89 | 1.96 | 2.09 | 1.99 | 2.39 | 4.08 | 0.99 |
| ***Ls*AA9** | | | | | | | | |
| **1** | (S=0) | 1.82 | 1.92 | 2.09 | 1.97 | 2.59 | 1.58 | 3.45 |
| $\mathbf{TS^{I}}$ | (S=0) | 1.84 | 1.93 | 2.06 | 1.93 | 2.50 | 2.15 | 1.45 |
| **2** | (S=0) | 1.89 | 1.97 | 2.06 | 1.99 | 2.22 | 1.78 | 1.00 |

**Additional discussion of reaction I:** It should be noted that in the first part of the reaction for *Ta*AA9 (Fig. 4c) the hydrogen bond between the $\text{O}-\text{H}_{\text{Tyr175}}$ and the second-sphere Gln173 is broken with a low activation energy of 29 kJ/mol. In a second step, the hydrogen of the tyrosine hydroxy group is transferred to $\text{O}_{\text{ox}}$. It was possible to obtain a stable intermediate "$\mathbf{Int^{I}}$" (we could ony obtain this intermediate in the triplet state and it was not obtained for *Ls*AA9). Since the energy of this intermediates is essentially the same as the transition state, we decided not to include it in Fig. 3 or Fig. 4 (this does not change any conclusions). The corresponding figures with "$\mathbf{Int^{I}}$" included are shown here, see Fig. S1 and S2. The latter Figure also includes a stable conformer of the tyrosine (**2′**), shown in Fig. S2, not shown in the main paper.

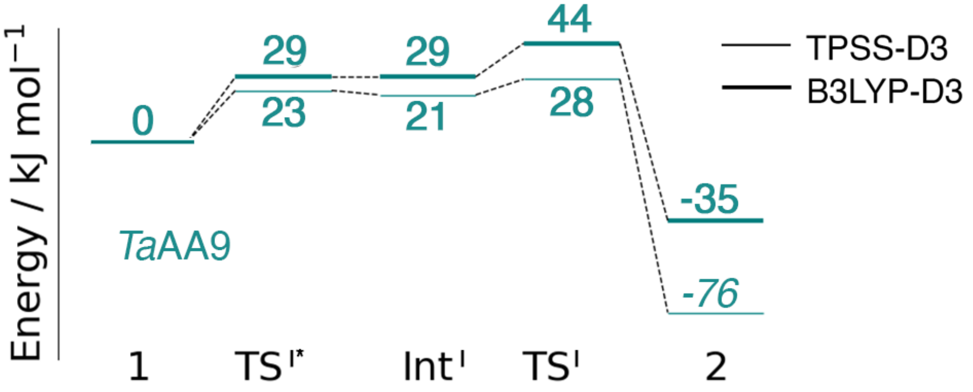


Figure S1: Energy diagram (in kJ/mol) for the reaction **I** for *TaAA9*. The reactant **1** in the triplet state was used as the reference. Energies were obtained employing def2-TZVPP based on structures optimized with TPSS/def2-SV(P). Results in bold style were obtained for the triplet state and those in italic were obtained for the open-shell singlet state.

This conformer differs from **2** in the orientation of the hydroxyl group, i.e., **2** and **2′** are related through a rotation of the $Cu{-}O_{hyd}$ bond. The energy difference between the isomers is rather low (20 kJ/mol) with the intermediate **2** forming a hydrogen bond between the the $OH_{hyd}$ of the $[CuOH]^+$ moiety and Gln173 being more stable. The spin-state splittings are rather functional dependent for both isomers. While B3LYP shows small splittings of 0.2-8.9 kJ/mol, TPSS predicts larger splittings of 19.9-20.6 kJ/mol depending on the isomer. Both isomers only show small changes for the spin densities and are therefore expected to have a similar electronic structure.

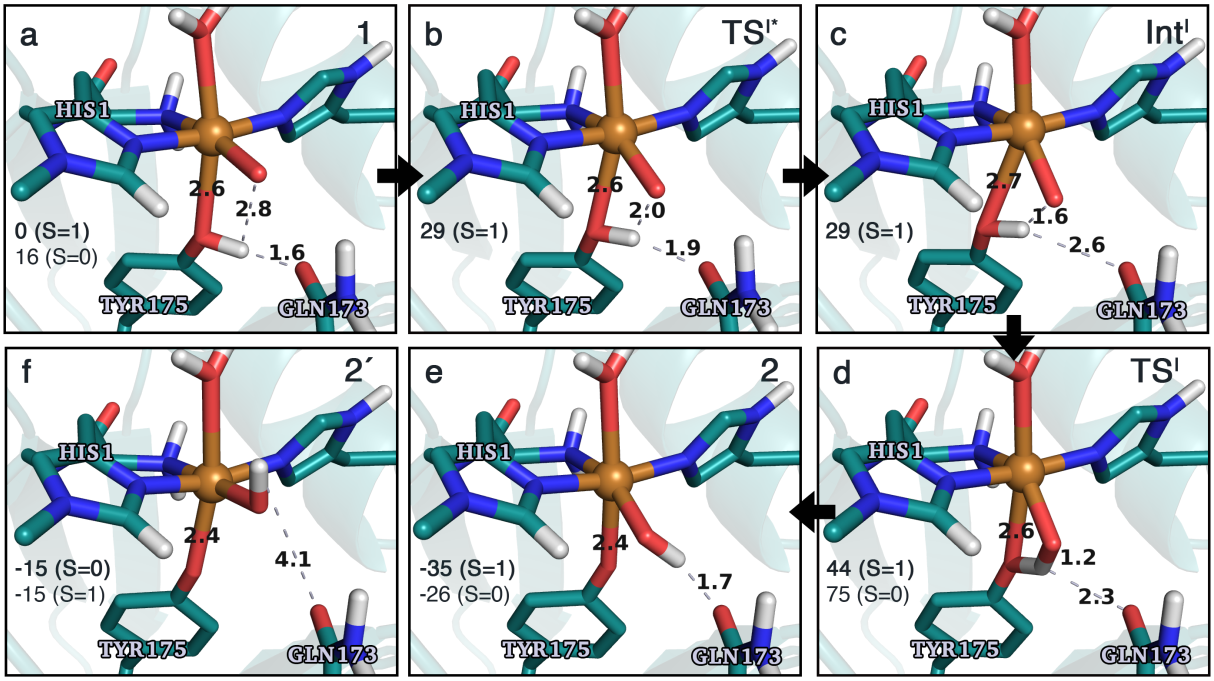


Figure S2: The hydrogen-abstraction reaction **I** illustrated for *Ta*AA9 (**a-e**). An alternative conformation (**2′**) of the tyrosyl **2** is shown in **f**. The structures were optimized using TPSS/def2-SV(P) while the energies shown are obtained employing B3LYP/def2-TZVPP. Only the structures for the most stable electron configuration (either open-shell singlet or triplet) is shown. Key distances are given in Å and energies in kJ/mol with reference to reactant **1** in the triplet state.

**Energies and distances of all states associated with reaction II:**

Table S3: QM/MM energies for *Ta*AA9 and *Ls*AA9 in reference to the reactant $[CuO]^+$ (**1**) in the triplet state. Energies for *Ta*AA9 were obtained with def2-TZVPP based on structures optimized with TPSS/def2-SV(P). Energies for *Ls*AA9 were taken from ref. 35.

| Intermediate | | Energy difference TPSS | B3LYP |
|---|---|---|---|
| ***Ta*AA9** | | | |
| **1** | (S=1) | 0.0 | 0.0 |
| **1** | (S=0) | 17.0 | 16.0 |
| $\mathbf{TS^{II}}$ | (S=1) | 81.7 | 98.0 |
| $\mathbf{TS^{II}}$ | (S=0) | 84.3[a] | 124.7[a] |
| $\mathbf{TS^{II}_{His86}}$ | (S=1) | 109.2 | 125.2 |
| **3** | (S=1) | 44.5 | 54.6 |
| **3′** | (S=1) | 56.6 | 74.2 |
| **3′** | (S=0) | 59.6 | 77.3 |
| $\mathbf{3_{His86}}$ | (S=1) | 63.3 | 75.3 |
| ***Ls*AA9** | | | |
| **1** | (S=1) | 0 | 0 |
| **1** | (S=0) | 12 | 13 |
| $\mathbf{TS^{II}}$ | (S=1) | 83 | 93 |
| $\mathbf{TS^{II}}$ | (S=0) | 88 | 97 |
| $\mathbf{TS^{II}_{His78}}$ | (S=1) | 91 | 100 |
| $\mathbf{TS^{II}_{His78}}$ | (S=0) | 91 | 106 |
| **3** | (S=1) | 52 | 52 |
| **3** | (S=0) | 47 | 70 |
| $\mathbf{3_{His78}}$ | (S=1) | 43 | 45 |
| $\mathbf{3_{His78}}$ | (S=0) | 39 | 44 |

[a] Calculations collapse into closed-shell singlets close to the TS (here, last proper open-shell singlet energies are given as $\mathbf{TS^{II}}$ for $S = 0$).

Table S4: Atom distances for chosen atoms in *Ta*AA9 in Å for reaction **II**. Structures were optimized using TPSS/def2-SV(P).

| Intermediate | | Distance Cu-$O_{Oxyl}$ | Cu-$N^{\delta}_{His1}$ | Cu-$N_{His1}$ | Cu-$N^{\epsilon}_{His86}$ | Cu-$O_{Tyr175}$ | $H^{\epsilon1}_{His1/H86}$-$O_{Oxyl}$ |
|---|---|---|---|---|---|---|---|
| ***Ta*AA9** | | | | | | | |
| **1** | (S=1) | 1.89 | 1.99 | 2.18 | 2.02 | 2.63 | 2.48/3.39 |
| $\mathbf{TS^{II}}$ | (S=1) | 1.96 | 1.95 | 2.15 | 2.02 | 2.61 | 1.20 |
| $\mathbf{TS^{II}_{His86}}$ | (S=1) | 1.96 | 1.98 | 2.09 | 1.95 | 2.75 | 1.20 |
| **3** | (S=1) | 1.98 | 1.93 | 2.12 | 2.03 | 2.72 | 0.99 |
| $\mathbf{3_{His86}}$ | (S=1) | 1.99 | 1.98 | 2.09 | 1.91 | 2.57 | 0.99 |

**Energies and distances of all states associated with reaction III:**

Table S5: QM/MM energies (in kJ/mol) calculated with the *smaller* QM region for *Ta*AA9 and *Ls*AA9 for reaction **III**. The energies are in reference to the respective reactant in the triplet state (**3′** and **3** for *Ta*AA9 and *Ls*AA9, respectively). Energies were obtained with def2-TZVPP based on structures optimized with TPSS/def2-SV(P). All energies for the larger QM regions can be obtained directly from Fig. 7 of the main paper.

| | | **Energy difference** | |
|---|---|---|---|
| **Intermediate** | | TPSS | B3LYP |
| ***Ta*AA9** | | | |
| **3′** | (S=1) | 0.0 | 0.0 |
| **3′** | (S=0) | 3.0 | 3.1 |
| **3** | (S=1) | -12.1 | -19.4 |
| $\mathbf{TS^{III}}$ | (S=1) | 81.3[a] | 100.5[a] |
| $\mathbf{TS^{III}}$ | (S=0) | 87.3[a] | 107.0[a] |
| $\mathbf{TS^{III}_{His86}}$ | (S=1) | -[b] | -[b] |
| **2** | (S=1) | -112.6 | -109.1 |
| **2** | (S=0) | -132.5 | -100.2 |
| ***Ls*AA9** | | | |
| **3** | (S=1) | 0.0 | 0.0 |
| **3** | (S=0) | -5.2 | -2.7 |
| $\mathbf{TS^{III}}$ | (S=1) | 123.5[a] | 143.8[a] |
| $\mathbf{TS^{III}}$ | (S=0) | -[b] | -[b] |
| $\mathbf{TS^{III}_{His78}}$ | (S=1) | 95.9 | 114.9 |
| **2** | (S=1) | -122.0 | -95.8 |
| **2** | (S=0) | -123.6 | -94.4 |

[a] Energies include high MM energies (>33 kJ/mol)

[b] Reaction ended in spontaneous rebound of the $O_{hyd}$ group from the Cu with the histidyl

Table S6: Atom distances for chosen atoms in Å for reaction **III**. The structures were optimized with the *bigger* QM region (including Phe43 and Pro30 for *Ta*AA9 and *Ls*AA9, respectively) employing TPSS/def2-SV(P).

| | | **Distance** | | | | | |
|---|---|---|---|---|---|---|---|
| **Intermediate** | | Cu-$O_{ox}$ | Cu-$N^{\delta}_{His1}$ | Cu-$N_{His1}$ | Cu-$N^{\epsilon}_{His86/78}$ | Cu-$O_{Tyr}$ | $C^{\epsilon1}_{His1/78}$-$H^{OH}_{Tyr}$ |
| ***Ta*AA9** | | | | | | | |
| **3′** | (S=1) | 1.97 | 1.98 | 2.16 | 2.01 | 2.74 | 2.32 |
| $\mathbf{TS^{III}}$ | (S=1) | 1.99 | 1.93 | 2.11 | 1.97 | 2.88 | 1.32 |
| **2** | (S=1) | 1.91 | 2.01 | 2.11 | 2.05 | 2.42 | 1.09 |
| ***Ls*AA9** | | | | | | | |
| **3** | (S=1) | 2.01 | 1.91 | 2.13 | 2.00 | 2.49 | 4.17 |
| $\mathbf{TS^{III}}$ | (S=1) | 2.03 | 1.89 | 2.10 | 1.93 | 2.63 | 1.41 |
| **2** | (S=1) | 1.95 | 1.98 | 2.14 | 2.00 | 2.18 | 1.09 |

**Mulliken spin densities:**

Table S7: Mulliken spin densities for chosen intermediates. If not further specified triplet spin densities are reported. Labelling of tyrosine and histidine atoms follows PDB labels. All values are obtained with B3LYP/def2-TZVPP.

| Complex | $[CuO(H)]^+$ | | His1 | | | | Tyrosine | | | | | | |
|---|---|---|---|---|---|---|---|---|---|---|---|---|---|
| Atom | Cu | $O_{ox/hyd}$ | N | $N^{\delta1}$ | $C^{\epsilon1}$ | $C^{\delta2}$ | $O_{Tyr}$ | $C^{\zeta}$ | $C^{\epsilon1}$ | $C^{\delta1}$ | $C^{\gamma}$ | $C^{\delta2}$ | $C^{\epsilon2}$ |
| **1** (*Ta*AA9) | 0.57 | 1.22 | 0.05 | 0.08 | 0.00 | 0.00 | 0.00 | 0.00 | 0.00 | 0.00 | 0.00 | 0.00 | 0.00 |
| **1** (*Ta*AA9, S=0) | -0.60 | 0.80 | -0.06 | -0.07 | 0.00 | 0.00 | 0.00 | 0.00 | 0.00 | 0.00 | 0.00 | 0.00 | 0.00 |
| **1** (*Ls*AA9) | 0.60 | 1.16 | 0.07 | 0.09 | 0.00 | 0.00 | 0.00 | 0.00 | 0.00 | 0.00 | 0.00 | 0.00 | 0.00 |
| **1** (*Ls*AA9, S=0) | 0.57 | -0.73 | 0.07 | 0.04 | 0.00 | 0.00 | 0.00 | 0.00 | 0.00 | 0.00 | 0.00 | 0.00 | 0.00 |
| **2** (*Ta*AA9) | 0.64 | 0.50 | 0.07 | 0.08 | 0.00 | 0.00 | 0.31 | -0.06 | 0.18 | -0.09 | 0.23 | -0.09 | 0.19 |
| **2** (*Ta*AA9, S=0) | 0.40 | 0.00 | 0.01 | 0.05 | 0.00 | 0.00 | -0.23 | 0.03 | -0.13 | 0.06 | -0.17 | 0.07 | -0.14 |
| **2** (*Ls*AA9) | 0.70 | 0.20 | 0.08 | 0.09 | 0.00 | 0.00 | 0.39 | -0.05 | 0.19 | -0.09 | 0.26 | -0.09 | 0.21 |
| **2** (*Ls*AA9, S=0) | 0.53 | 0.10 | 0.03 | 0.08 | 0.00 | 0.00 | -0.37 | 0.04 | -0.19 | 0.09 | -0.27 | 0.09 | -0.20 |
| **2′** (*Ta*AA9) | 0.61 | 0.35 | 0.07 | 0.08 | 0.00 | 0.00 | 0.36 | -0.05 | 0.22 | -0.11 | 0.28 | -0.11 | 0.22 |
| **3** (*Ta*AA9) | 0.61 | 0.50 | 0.08 | 0.14 | 0.56 | 0.07 | 0.00 | 0.00 | 0.00 | 0.00 | 0.00 | 0.00 | 0.00 |
| **3** (*Ls*AA9) | 0.65 | 0.35 | 0.08 | 0.02 | 0.73 | 0.03 | 0.00 | 0.00 | 0.00 | 0.00 | 0.00 | 0.00 | 0.00 |
| **3′** (*Ta*AA9) | 0.60 | 0.56 | 0.07 | 0.10 | 0.54 | 0.07 | 0.00 | 0.00 | 0.00 | 0.00 | 0.00 | 0.00 | 0.00 |

Table S8: Mulliken spin densities for chosen intermediates. If not further specified triplet spin densities are reported. Labelling of tyrosine and histidine atoms follows PDB labels. All values are obtained with TPSS/def2-TZVPP.

| Complex | $[CuO(H)]^+$ | | His1 | | | | Tyrosine | | | | | | |
|---|---|---|---|---|---|---|---|---|---|---|---|---|---|
| Atom | Cu | $O_{ox/hyd}$ | N | $N^{\delta1}$ | $C^{\epsilon1}$ | $C^{\delta2}$ | $O_{Tyr}$ | $C^{\zeta}$ | $C^{\epsilon1}$ | $C^{\delta1}$ | $C^{\gamma}$ | $C^{\delta2}$ | $C^{\epsilon2}$ |
| **1** (*Ta*AA9) | 0.54 | 1.22 | 0.05 | 0.08 | 0.00 | 0.00 | 0.00 | 0.00 | 0.00 | 0.00 | 0.00 | 0.00 | 0.00 |
| **1** (*Ta*AA9, S=0) | -0.48 | 0.69 | -0.06 | -0.07 | 0.00 | 0.00 | 0.00 | 0.00 | 0.00 | 0.00 | 0.00 | 0.00 | 0.00 |
| **1** (*Ls*AA9) | 0.56 | 1.15 | 0.07 | 0.09 | 0.00 | 0.00 | 0.00 | 0.00 | 0.00 | 0.00 | 0.00 | 0.00 | 0.00 |
| **1** (*Ls*AA9, S=0) | -0.07 | 0.08 | -0.01 | 0.02 | 0.00 | 0.00 | 0.00 | 0.00 | 0.00 | 0.00 | 0.00 | 0.00 | 0.00 |
| **2** (*Ta*AA9) | 0.61 | 0.55 | 0.08 | 0.09 | 0.00 | 0.00 | 0.25 | -0.03 | 0.16 | -0.07 | 0.20 | -0.07 | 0.16 |
| **2** (*Ta*AA9, S=0) | 0.19 | 0.08 | 0.00 | 0.04 | 0.00 | 0.00 | -0.15 | 0.00 | -0.09 | 0.04 | -0.13 | 0.04 | -0.10 |
| **2** (*Ls*AA9) | 0.72 | 0.25 | 0.10 | 0.10 | 0.00 | 0.00 | 0.30 | -0.02 | 0.15 | -0.07 | 0.22 | -0.07 | 0.17 |
| **2** (*Ls*AA9, S=0) | 0.30 | 0.08 | 0.00 | 0.08 | 0.00 | 0.00 | -0.24 | 0.01 | -0.13 | 0.05 | -0.19 | 0.06 | -0.14 |
| **2′** (*Ta*AA9) | 0.60 | 0.44 | 0.08 | 0.08 | 0.00 | 0.00 | 0.28 | -0.02 | 0.18 | -0.08 | 0.23 | -0.08 | 0.18 |
| **3** (*Ta*AA9) | 0.52 | 0.55 | 0.09 | 0.14 | 0.56 | 0.08 | 0.00 | 0.00 | 0.00 | 0.00 | 0.00 | 0.00 | 0.00 |
| **3** (*Ls*AA9) | 0.58 | 0.35 | 0.10 | 0.02 | 0.71 | 0.03 | 0.00 | 0.00 | 0.00 | 0.00 | 0.00 | 0.00 | 0.00 |
| **3′** (*Ta*AA9) | 0.53 | 0.58 | 0.09 | 0.11 | 0.55 | 0.08 | 0.00 | 0.00 | 0.00 | 0.00 | 0.00 | 0.00 | 0.00 |

## UV-vis spectra

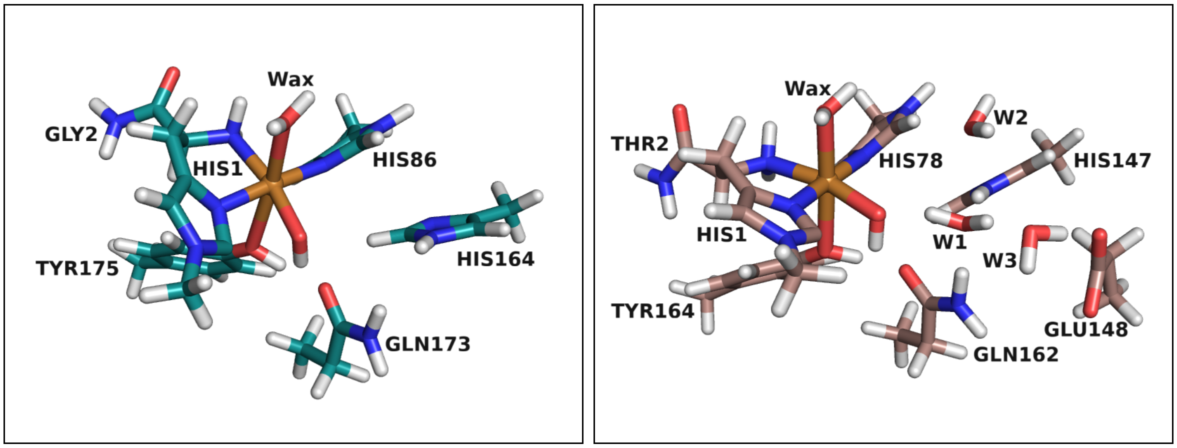


Figure S3: QM regions for *Ta*AA9 (left) and *Ls*AA9 (right) employed for the TD-DFT calculations (histidyl **3** as example). The structures were QM/MM optimized using TPSS/def2-SV(P). The TD-DFT structures were slightly truncated compared to the QM regions used for the QM/MM calculations (see computational details). Labels refer to PDB 2YET[22] and 5ACF[47] for *Ta*AA9A and *Ls*AA9, respectively.

**Tyrosyl intermediates:** The here calculated spectra for the tyrosyl **2** for *Ta*AA9 are shown in Fig. S4 together with the corresponding spectra for *Ls*AA9 from ref. 40. To evaluate the effects of the truncation of the QM region and the protein environment on the TD-DFT spectra, we additionally calculated the spectrum for the tyrosyl **2** for *Ta*AA9 with surrounding point charges (obtained from the QM/MM calculation) and for the untruncated QM region as shown in Fig. 2. Fig. S5 shows that the truncation and inclusion of protein environment leads to two intense transitions at 348 nm (3.57 eV) and 359 nm (3.45 eV) that agree well with the most intense transition in the vacuum spectrum at 357 nm (3.48 eV). An analysis of the major orbital contributions of the most intense peaks of the triplet and open-shell singlet state show a large involvement of the tyrosine orbitals. In good agreement to the nature of the transitions of *Ls*AA9,[40] the metal-to-ligand charge transfer (MLCT) excitations involve mainly transitions from orbitals of Cu d-character to an orbital of $\pi$-character on tyrosine. The intense peak for the triplet state at 656 nm (1.89 eV) does not occur in the spectrum of the triplet of *Ls*AA9 and occurs mainly due to transitions from

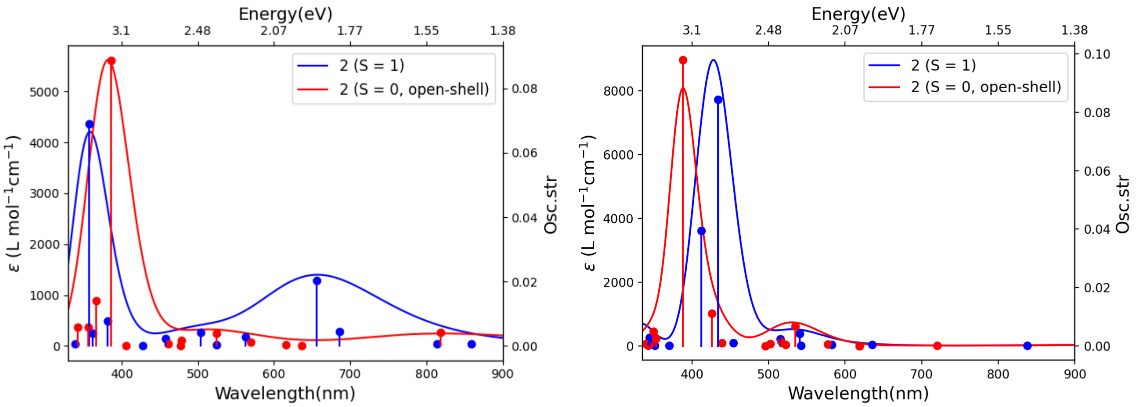


Figure S4: Calculated UV-vis spectra of **2** for *Ta*AA9 (left) and *Ls*AA9 from ref.[40] (right). The spectra were obtained using CAM-B3LYP/def2-TZVPP.

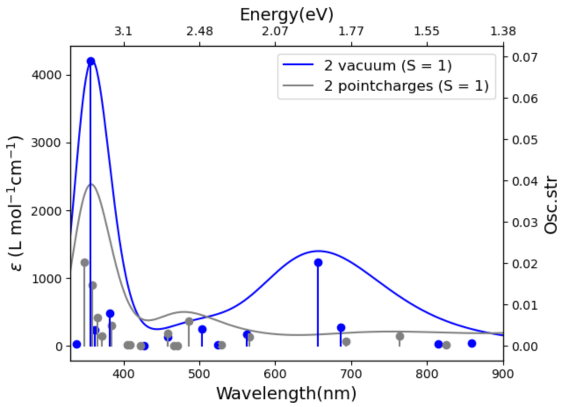


Figure S5: Calculated UV-vis spectra of **2** for *Ta*AA9 in the triplet spin state. The spectra were obtained using CAM-B3LYP/def2-TZVPP in vacuum or with point charges (the same charges as used in the QM/MM calculations). The spectra with point charges was calculated for the untruncated QM region as shown in Fig. 2.

orbitals of hydroxyl lone pair character to orbitals of $\pi$-character on tyrosine. We note that the spectrum of the tyrosyl isomer **2′** (not shown) of *Ta*AA9 does not show this peak, while otherwise showing almost identical features to the spectrum of intermediate **2**. Moreover, this transition is also not present if the spectrum is calculated with pointcharges (see Fig. S5).

**Histidyl intermediates:** The calculated spectra for the histidyl intermediates are shown in Fig. S6 (*Ls*AA9) and S7 (*Ta*AA9). The former figure additionally shows the spectrum where the histidyl radical is formed with His78. In case of *Ta*AA9, we could only obtain the triplet of **3**, whereas for the isomer **3′**, both open-shell and triplet spectra were calculated. The most intense peak of the histidyl **3** of *Ls*AA9 is at 427 nm (2.91 eV) for the

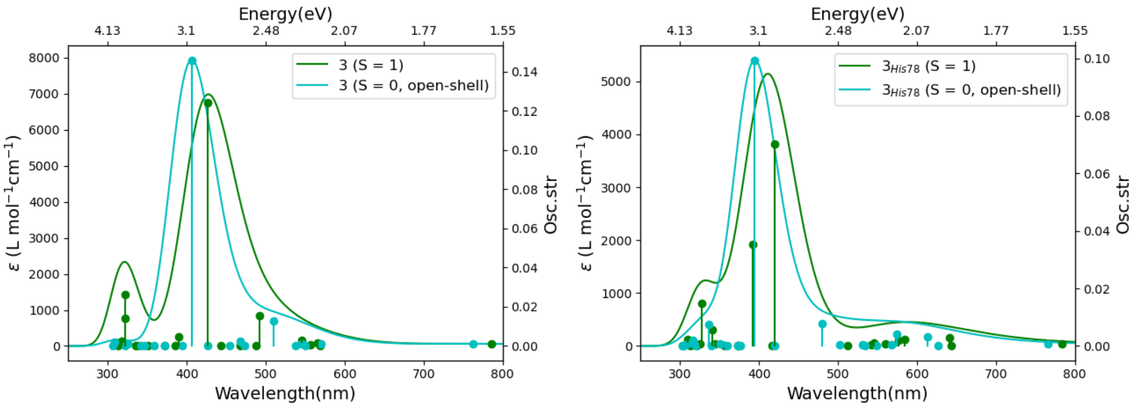


Figure S6: Calculated UV-vis spectra for **3** (left) and $\mathbf{3_{His78}}$ (right) of *Ls*AA9. The spectra were obtained using CAM-B3LYP/def2-TZVPP. The structures were obtained from the QM/MM calculations employing TPSS/def2-SV(P) with the *smaller* QM region not including Pro30.

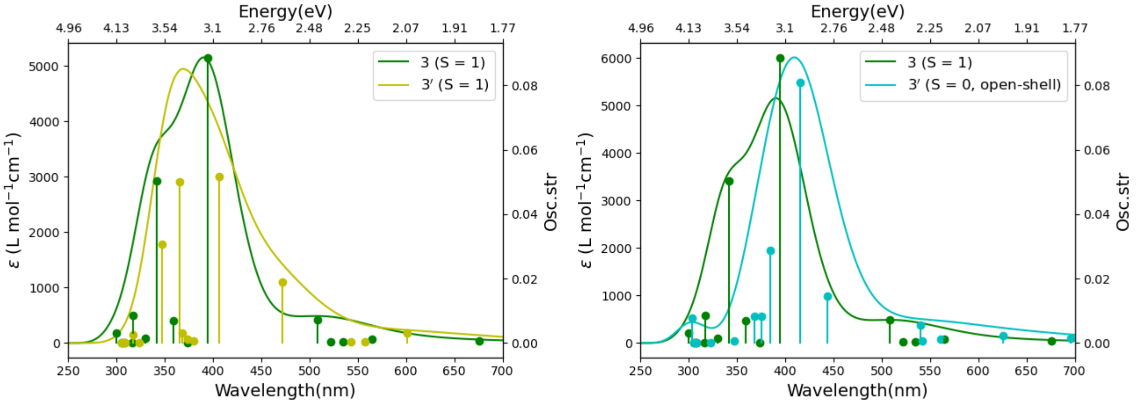


Figure S7: Calculated UV-vis spectra for the histidyl in *Ta*AA9. Comparison of **3** and **3′** in the triplet state (left) and comparison of the triplet state of **3** with the open-shell singlet state of **3′** (right). The spectra were obtained using CAM-B3LYP/def2-TZVPP. The structures were obtained from the QM/MM calculations employing TPSS/def2-SV(P) with the *smaller* QM region not including Phe43.

triplet state and 406 nm (3.05 eV) for the open-shell singlet state. Both occur mainly due to transitions from orbitals of hydroxyl lone pair character to an orbital of $\pi$-character on the histidyl His1. The triplet state spectrum also shows a less intense peak at higher energies 322 nm (3.86 eV). The transition shows an involvement of the histidyl with a ligand-to-metal charge transfer nature. However, the transition is highly delocalized. The intermediate with a histidyl on $\mathbf{3_{His78}}$ overall shows a similar absorption spectrum: the most intense peak is

at 420 nm (2.95 eV) for the triplet state and 395 nm (3.14 eV) for the open-shell singlet and both are of the same nature as the most intense peak of intermediate **3**. The triplet state shows two less intense peaks at higher energies at 392 nm (3.16 eV) and 294 nm (4.22 eV) with a highly delocalized nature of the transitions.

Intermediate **3** for *Ta*AA9 also shows the intense feature around 400 nm seen for **3** in the *Ls*AA9 spectrum. However, an additional transition is also seen at higher energy: the two most intense peaks in Fig. S7 (green spectrum, left) are at 342 nm (3.63 eV) and 395 nm (3.14 eV). An analysis of the major orbital contributions of these peaks show a large involvement of the histidyl. The main peak at 395 nm mainly involves transitions from orbitals of hydroxyl lone pair character to an orbital of $\pi$-character on the histidyl His1. The less intense peak at higher energies (342 nm) occurs due to ligand-to-metal charge transfer transitions including orbitals on the histidyl. Similar transitions can also be observed for the isomer $\mathbf{3'}$, albeit with the two most intense peaks red-shifted to 348 nm (3.57 eV) and 406 nm (3.05 eV) for the triplet state and 384 nm (3.23 eV) and 416 nm (2.98 eV) for the open-shell singlet. Overall, the spectra of **3** and $\mathbf{3'}$ are quite similar.
